# Effective tools for RNA-derived therapeutics: siRNA interference or miRNA mimicry

**DOI:** 10.7150/thno.62642

**Published:** 2021-08-11

**Authors:** Peipei Wang, Yue Zhou, Arthur M. Richards

**Affiliations:** 1 Cardiovascular Research Institute, Yong Loo Lin School of Medicine, National University of Singapore, 117599 Singapore.; 2 Department of Medicine, National University Health System, 119228 Singapore.; 3 Christchurch Heart Institute, Department of Medicine, University of Otago Christchurch, New Zealand.

**Keywords:** RNA interference (RNAi), therapeutics, siRNA, miRNA, off-target effect

## Abstract

The approval of the first small interfering RNA (siRNA) drug Patisiran by FDA in 2018 marks a new era of RNA interference (RNAi) therapeutics. MicroRNAs (miRNA), an important post-transcriptional gene regulator, are also the subject of both basic research and clinical trials. Both siRNA and miRNA mimics are ~21 nucleotides RNA duplexes inducing mRNA silencing. Given the well performance of siRNA, researchers ask whether miRNA mimics are unnecessary or developed siRNA technology can pave the way for the emergence of miRNA mimic drugs. Through comprehensive comparison of siRNA and miRNA, we focus on (1) the common features and lessons learnt from the success of siRNAs; (2) the unique characteristics of miRNA that potentially offer additional therapeutic advantages and opportunities; (3) key areas of ongoing research that will contribute to clinical application of miRNA mimics. In conclusion, miRNA mimics have unique properties and advantages which cannot be fully matched by siRNA in clinical applications. MiRNAs are endogenous molecules and the gene silencing effects of miRNA mimics can be regulated or buffered to ameliorate or eliminate off-target effects. An in-depth understanding of the differences between siRNA and miRNA mimics will facilitate the development of miRNA mimic drugs.

## Introduction

In 1998, Fire and Mello first reported RNA interference (RNAi), gene silencing by double-stranded RNA (dsRNA) 1. This work won the Nobel Prize in 2006 and RNAi was judged to be “a fundamental mechanism for controlling the flow of genetic information in cells”. The first miRNA was discovered by Lee *et al.* from *C elegans* in 1993 2. They confirmed that *lin-4* RNAs could regulate translation of the gene *Lin-14* through an antisense mechanism. However, miRNAs did not attract much attention until they were reported to be also present in humans and many other species 7 years later 3. Like dsRNAs, miRNAs downregulate gene expression. DsRNA drugs belong to oligonucleotide-based therapeutics, which have become the third major drug development platform alongside small molecules and protein-based biologics.

There are two important approaches in oligonucleotide-based therapeutics, single stranded antisense oligonucleotides (ASO) and dsRNAs. ASOs are single stranded DNA sequences which form DNA-RNA heteroduplex with mRNA and lead to RNA degradation by activating RNase H or by altering splicing or inhibit translation 4, 5. The second major avenue is miRNA inhibitors, also called antagomiRs, which irreversibly bind to miRNAs to block their function 6, 7. On the other hand, miRNA mimics are miRNA-like dsRNAs. As the mechanisms and drug development of ASOs differ from those for dsRNAs, they are not included in this review. Both siRNA and miRNA mimics are dsRNA ~21 nt in length. To date, three siRNA drugs to treat porphyria, Transthyretin (TTR) amyloidosis and very recently hyperlipidemia have been approved by FDA for clinical use. A number of further siRNAs, but only three miRNA mimics, have entered into clinical trials. Each successful RNAi drug development follows a similar trajectory of accrued basic science and technological advance 8. Following the lead of successful development of siRNA therapeutics, common challenges in miRNA mimic drug development: manufacturing, stability and delivery, can be addressed by increasingly mature technologies. This history is well reviewed and discussed elsewhere 9, 10. This review provides a comprehensive comparison between siRNAs and miRNA mimics, (1) to appreciate the common features of siRNA and miRNA and lessons that could be learnt from the successful development of siRNAs (2) to identify unique characteristics of miRNA that offer additional therapeutic advantages and opportunities and (3) reviewing areas of ongoing miRNA research that will contribute to the further understanding and clinical application of miRNA mimics. We hope to provide in-depth understanding of obstacles and advantages in the development of miRNA therapeutics.

## The basics of miRNA and siRNA

### Naturally existing *vs*. artificial biomolecules

**Figure 1:**
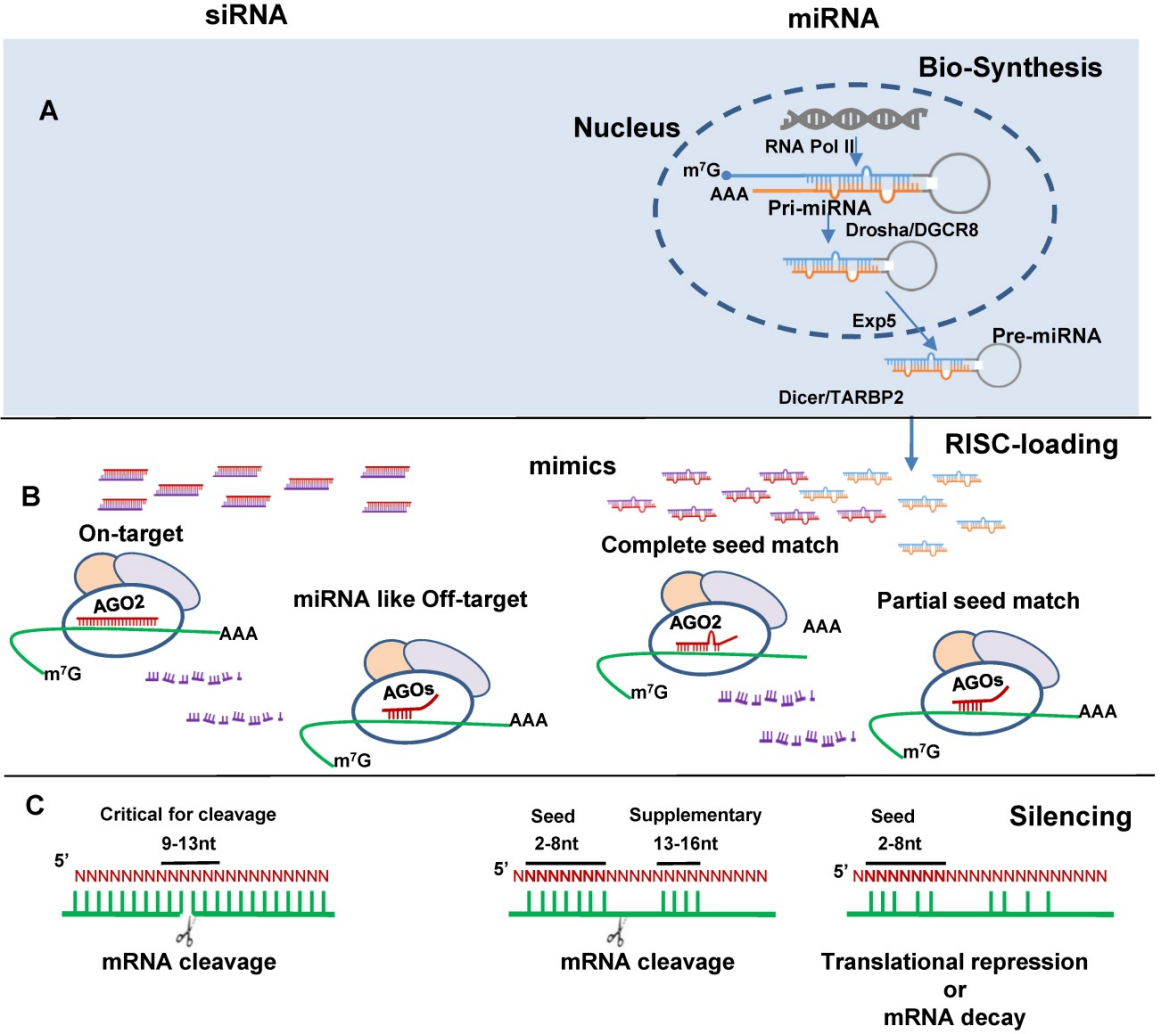
** SiRNA and miRNA mimics induce gene silencing. (A)** miRNA biogenesis including pri-miRNA formation from DNA transcription by RNA polymerase II (RNA Poly II), pre-miRNA formation by Drosha and the double-stranded RNA-binding protein DiGeorge critical region 8 (Drosha/DGCR8) processing, pre-miRNA exportation from nuclear to cytosol by the export receptor exportin 5 (Exp5), and mature miRNA formation by Dicer and RNA binding protein TARBP2 (Dicer/TARBP2) processing. **(B)** siRNA (left) and miRNA(right) RNA-induced silencing complex (RISC)-loading. SiRNA by design has perfect complementary binding to the target mRNA and miRNA with seed region complete complementary to binding site (BS) in the mRNA 3' untranslated region (3'-UTR) interact with AGO2. SiRNA partially complementary to the target mRNA and miRNA with partial seed match, interact with AGO1, 3, 4. Red color indicates siRNA antisense and miRNA mimic guide strands; purple color indicates siRNA sense and miRNA mimic passenger strands; blue and orange color indicates endogenous miRNA guide and passenger strands respectively. **(C)** Binding pattern directed target recognition leads to target cleavage or translational repression and mRNA decay.

MiRNA and siRNA differ greatly in their biogenesis and biological origins. MiRNAs are indispensable endogenous post-transcriptional gene regulators. Global loss of miRNAs is lethal 11, 12. They are highly conserved across species, ubiquitously expressed in various tissues and involved in all kinds of cellular processes. It is estimated 60-90% of human protein-coding genes are regulated by miRNAs 13, 14. MiRBase currently registers 38,589 miRNA entries across 271 organisms; amongst them 2,675 human miRNAs 15-17. MiRNA biogenesis have been well reviewed periodically 18-20. In brief, they are initially expressed as pri-miRNA transcripts (can be >1,000 nt) and then processed by Drosha/DGCR8 into pre-miRNA hairpins (~70 nt). The pre-miRNAs are transported from nucleus to cytoplasm by Exportin-5 where they are further processed by Dicer/TARBP2 into miRNA duplex (Figure 1A). Dysregulation by disease exhibits unique disease-specific patterns. Therefore, to identify and correct these disruptions renders miRNAs as candidate diagnostic biomarkers and the target of therapeutic interventions. Drosha or Dicer non-independent miRNA biogenesis, known as non-canonical pathway, has also been reported which generates less than 1% of conserved miRNAs 19.

SiRNAs are not naturally expressed in humans and other mammals. The only exceptions are found in murine germline cells 21, 22. Endogenous siRNAs (endo-siRNAs) were discovered to be present in plants and invertebrates 1, 23 and zebrafish 24. Endo-siRNAs can be generated from convergent transcripts, sense-antisense pairs, gene/pseudogene duplexes or repeat-associated transcripts from centromeres and transposons 25. Viral infection can also lead to siRNA generation as viruses are a source of long dsRNAs from viral genomic replication 26. Biogenesis of the endo-siRNAs involves RNA-dependent RNA polymerase and a siRNA-specific isoform of Dicer. These enzymes are not found in mammals 27. Loss-of-function studies in *C elegans* and *drosophila* indicate that endo-siRNA functions as a viral defense mechanism 27, 28. In mammals, a similar defense function is carried out by the interferon system 29, 30.

MiRNA mimics and siRNAs have many features in common. They are dsRNA ~21 nt in length, bind to Argonaute protein (AGO) to form an RNA-induced silencing complex (RISC) and induce target gene silencing (Figure 1B). The mechanism through which miRNAs or siRNAs are integrated with AGOs is not fully understood. Data from single strand loading models suggest dsRNAs are unwound before loading. However, strong evidence supports a duplex-loading model, by which dsRNA duplexes are incorporated into AGOs followed by dissociation and degradation of the passenger or sense strand 31. X-ray crystallography structural analysis reveal that miRNA guide and siRNA antisense strands bind to human AGO2 in RISC 32, 33. For miRNA AGO loading, Dicer cleaved double-stranded miRNA may directly transfer to AGO 34. AGO loading is asymmetrical in that the strand with lower thermostability at the 5' end is preferentially selected for loading 35, 36. Loaded strands are protected from degradation by making it inaccessible to endogenous nucleases and the other stands are degraded 37. The guide/antisense strand then directs the RISC complex to target mRNA via Watson-Crick base-pairing 38. SiRNA is designed to be perfectly complementary to the target mRNA and, miRNA follows the “seed-pairing rule”, a complementary binding of miRNA seed region to binding site (BS) located in the mRNA 3' untranslated region (3'-UTR) (Figure 1C). The seed region involves nt 2-8 from miRNA 5' end or possibly nt 2-7 and 2-6. In addition, supplemental region in the 3' half of miRNAs, particularly nt 13-16, can also be involved in target recognition 39. Recent work demonstrated that the supplemental region is important in directing miRNAs with same seed sequence to bind to different targets 40. There are 4 types of AGO1-4 capable of loading dsRNA. AGO2 is the only one interacts with siRNA or those miRNAs having seed region with perfect or near perfect complementary sequence to mRNAs to induce mRNA cleavage 41, 42. All AGOs could induce gene silencing via translational repression and mRNA decay. These mechanisms have been reviewed elsewhere 43.

### MiRNA turn-over and RISC loading

The expression of miRNA is precisely controlled in a spatial and temporal manner. Using thiol-linked alkylation (4sU labelling) for the metabolic sequencing of small RNA (SLAMseq), Reichholf *et al.* reported that miRNA is the most rapidly synthesized form of cellular RNAs. In as short as 5, 15 and 30 min, the production of 4sU-labelled miRNAs are 43%, 69% and 90% of total miRNAs respectively in Drosophila S2 cells 44. Similar results have been reported from studies of mammalian cells 45. However, the assembly of miRNA into AGOs is slow. Notably, ~40% of miRNA duplexes may be non-specifically degraded before AGO-loading 44. The passenger strands are also degraded in the process of AGO-loading. Over-production of miRNA duplexes is believed to secure miRNA function by competing with other non-coding RNAs (rRNA, tRNA, snRNA and snoRNA) for access to AGOs. AGO2-enriched miRNAs are much more stable than AGO1-enriched miRNAs with half-lives >24h and 16h respectively, which affects miRNA turn-over 44.

The RISC-loading of individual miRNAs is specifically controlled by endogenous regulatory mechanisms. The endogenous ratio of RISC-loaded/total miRNA varies over a >100-fold range in human cell lines 46. The mechanisms are not fully elucidated but they are miRNA-specific and actively regulated. Endogenous miRNAs exert baseline effects which may buffer the effects of exogenous miRNA mimics. This is supported by a study of the dose-dependent effects, 0-66 nM, of five miRNA mimics on target silencing in HEK 293T cells 47. They correspond to five endogenous miRNAs identified with different levels of RISC-loaded abundance. With respect to the most endogenously abundant miR-20, mimics at different concentrations had no gene silencing effects at all, whereas for the least abundant miR-26, mimics yielded dose-dependent effects with a maximum of 80% target silencing. Endogenous counterparts and RISC loading capacity will modulate mimics effects. These observations may confer a favorable safety profile to miRNA replacement therapy, as loss of endogenous miRNA is specifically restricted to diseased tissue, making them responsive to mimic treatment, whilst healthy tissue may maintain homeostasis to buffer out on-target side-effects. Evidence of differential miRNA function in healthy vs. disease contexts will be reviewed in the next section.

## Synthetic siRNA and miRNA mimic design

### Synthetic siRNA

**Synthetic siRNA** by design is a tool for specific, robust knockdown of a single gene. More than 80% knockdown is commonly achieved in experimental and clinical applications 48. Chemically synthesized siRNAs are well-defined, easy to manufacture and amenable to extensive modifications. They are typically ~22 nt double-stranded duplexes with perfect Watson-Crick complementarity and two nt overhang at the 3' end to resemble Dicer cleavage products which facilitate AGO-loading. Among the 4 AGOs, AGO2 cleaves the designated target mRNA and is critical for robust siRNA mediated gene silencing. The cleavage is believed to start from the phosphodiester bond of the target mRNA that lies across the 10^th^ and 11^th^ nt of the siRNA antisense strand 49, 50. It is not a coincidence that siRNAs are of similar length to miRNA. Short dsRNAs (15 nt or less) lose RNAi activity and longer dsRNAs (>30 nt) activate protein kinase R (PKR) to stimulate innate immune responses 51, 52. Dicer substrate siRNA (25-27 nt), also called DsiRNAs, have shown increased potency over conventional 22 nt siRNAs, assessed by persistence of the antisense strand, better RISC loading and longer-lasting RNAi activity 53, 54. This is probably due to Dicer's role in RISC loading as it has been shown to directly bind to AGO and form the pre-RISC complex 55. However, DsiRNA has had limited development towards clinical application because dicer processing and nt chemical modifications confound each other 56. Single-stranded siRNA (ss-siRNA) require additional 5'-phosphate modification and showed greatly reduced potency compared to dsRNA 57-59 although more recent studies indicate that specific design strategies can improve ss-siRNA potency 60, 61. It remains an alternative option with the advantages of having no risk of sense strand misloading and better cellular uptake. Small hairpin RNAs (shRNAs) have been used to achieve RNAi as siRNA 62. Recently BCL11A shRNA to treat sickle cell disease has been reported into clinical trial 63. The advantages of shRNA are (1) viral vectors-based delivery for primary and non-dividing cells which are hard for transfection; (2) AAVs from episomes transfected into the host genome for stable expression. As this procedure requires nuclear transcription processing with the potential risk of over-saturating the miRNA biogenesis pathway, shRNA dose and sequence must be carefully optimized 64.

Sense strands of siRNAs are degraded. However, very rarely they can be misloaded into RISC which generates unintended off-target effects 65. Effective chemical modifications have been developed to avoid misloading or abrogate sense strand activity, such as 2'-O-methylation, 5'-O-methylation, 5'-Morpholino, 5' ligand conjugation 66-69. The principles guiding siRNA design are high potency, high metabolic stability, reduced off-target effects and elimination of immune stimulation. Strategies involve sequence optimization, chemical modifications and experimental screening 70, 71.

### MiRNA mimics

Endogenously, precursor miRNA hairpins are processed by Dicer/TARBP2 to generate one or two mature miRNAs, -5p and -3p from 5' and 3' termini respectively. They are different miRNAs with distinct seed sequences, target populations, functions and especially different expression levels 72-74. Exogenous miRNA generated via DNA/viral vectors, and synthetic miRNA precursors, do not offer clear therapeutic applications due to the uncontrolled expression of miRNA -3p and -5p strands. Single-stranded miRNA mimics have also been tested to show *in vitro* RNAi activity and cellular effects 75-77. They share the same pros and cons as ss-siRNAs and have not been well developed or widely used. In contrast, miRNA mimics, typically ~21-nt double-stranded duplexes, are well investigated in research and clinical contexts.

Compared to siRNAs, miRNA mimic design and chemical modification are relatively neglected topics among published reports. MiRNA mimics contain a guide strand with sequence identical to the endogenous miRNA -3p or -5p. Chemical modifications that have been developed in siRNAs for nuclease resistance, such as 2'-O-Me, 2'-F and phosphorothioate backbone linkage substitution, can be readily applied to the guide strand 78. Commercial miRNA mimic manufacturers typically synthesize the passenger strand according to miRBase sequence and use proprietary chemical modifications to inactivate it (https://www.thermofisher.com). Although design of the passenger strand is not adequately discussed, independent laboratory testing of mimics has generally shown correct selective loading with a low passenger:guide strand ratio (≤10-15%) 79. Mimics used in clinical trials are developed by individual RNAi drug companies and the rationale of design is often not fully apparent due to the protection of intellectual property (IP). For example, the passenger strands of 4 miR-15/16 family mimics have four 2′-O-methyl-modified nucleotides at each end 80. The passenger strand of miR-29b-3p mimic has 2'-O-Methyl modifications and is conjugated to cholesterol at 3' end to enhance cellular uptake 81.

To date, commercially available miRNA mimics have been the main source of mimics investigated in basic scientific research, reflecting their easy accessibility and ostensible strand selection control. The main sources of commercial miRNA mimics are summarized in Table 1. Although it is inevitably complicated by the need for commercial entities to preserve their IP, open information and communications between the academia and industry may benefit future studies. In any case, researchers need to be aware of the potential risk of misloading and the danger of relying solely upon manufacturers for quality control 82.

## siRNA off-target effects *vs.* miRNA mimic condition-dependent and synergized effects

### MiRNA-like off-target effects of siRNA

Sequence-specific miRNA-like unintended gene repression is the major cause of off-target effects for siRNAs, which affects a large number of genes. Wide-spread miRNA-like off-target effects of siRNAs were first documented in 2003 by two independent groups 83, 84. In this early work, 16 and 8 siRNAs were designed to silence *IGF1R* and *MAPK14* respectively. Besides the target gene knockdown, each set of siRNAs produced a distinct gene downregulation profile which does not dependent on the target gene knockdown. Sequence alignment demonstrated that siRNA could bind with off-target mRNAs through partial Watson-Crick complementarity. The binding regions distributed randomly throughout the siRNA 85. This is supported by an elegant study demonstrating that miRNA-like off-target effects of siRNAs are abrogated by (1) the sequestration of antisense strand but not sense strand; (2) replacing nt in the seed region; (3) thermal destabilization of seed binding with GNA to replace nt in the seed region or (4) the inhibition of RISC-loading by 5'-end capping of the siRNA antisense strand 86. An analysis of the human 3′-UTR database reveals that any random 7nt sequence can map to at least 17 different 3'-UTRs with complementary binding sites 87, 88. Substitution of nt within the siRNA seed region abolished the original array of off-target effects but generated new sets of silenced genes 85. Therefore, it is hard to predict and control the off-target effects of siRNAs.

SiRNA off-target effects can lead to toxic phenotypes such as reported cell death in Hela cells 89 and growth inhibition in multiple human and mouse cell lines 90. Two apolipoprotein B (APOB) siRNAs showed robust >95% reduction of liver *APOB* mRNA and serum APOB protein in mice. However, many additional genes were distinctly altered indicating off-target effects 91. In a preclinical study of Inclisiran (*PCSK9* siRNA), specific *PCSK9* mRNA cleavage was confirmed together with 73 specific gene sequences and additional 11 unrelated sequences retrieved from 5'RACE followed by cloning and sequencing. Acute hepatoxicity (>4-fold elevation of serum alanine transaminase (ALT) and aspartate aminotransferase (AST) was observed in 1 out of 9 cynomolgus monkeys which might be attributed to off-target effects 92. In a rat toxicology study of GalNAc-conjugated siRNAs, 5 of 8 randomly selected siRNAs showed significant hepatotoxicity as demonstrated by elevated ALT and histopathological findings of hepatocyte degeneration and liver fibrosis. Off-target effects were shown to be responsible for the hepatotoxicity, as RISC-loading blockade, ASO inhibition and seed region swapping mitigated the toxic effects 86. Due to this, ALN-AAT (alpha-1 antitrypsin) to treat AAT deficiency and Revusiran to treat amyloidosis was halted by Alnylam. Hence miRNA-like off-target effects remain a serious problem in siRNA therapeutics.

Great efforts have been made to tackle this problem. As the miRNA-like off target effect is concentration dependent, pooling siRNAs with same target specificity, thus diluting off-target effects from any one siRNA, is one option 93. However, results are inconsistent and this strategy can cause more off-target effects from additional siRNAs. Chemical modification is more practical. The 2′-*O*-methyl ribosyl substitution at position 2 from 5' end in the antisense strand reduced mean off-target silenced genes from ~40% to ~20%. However, important off-target gene regulation persists, as this modification is ineffective in the presence of strong seed-region binding energies 94. More aggressive approaches include incorporation of locked and unlocked nucleic acid (LNA and UNA) 95, a “bulge” 96 or substitution of position 6 with basic spacers in the antisense strand 97. These methods generally work by destabilizing the RNA-RNA interaction at the seed region 98, 99 and thus inevitably carry the risk of sacrificing some strength of on-target binding 100. Furthermore, off-target effects are species-specific, as mouse liver and cell line showed consistency, whilst mouse *vs* human cell lines exhibited little overlap in off-target gene regulation profiles 101. This suggests that genomic background and transcriptome profile are key determinants of siRNA off-target effects. This significantly adds to the difficulty and cost of siRNA preclinical testing and drug development.

### Specific on-target side-effects of siRNA

As siRNA knockdown of the target gene is not organ-selective, on-target side effects can limit the application of siRNA therapeutics. Chemically stabilized and lipid nanoparticle delivered siRNAs both showed non-selective tissue distribution in rodents as assessed by imaging and mass spectrometry 102-105. SiRNA knocks down its target gene ubiquitously in all organs to which it is distributed. Systemic administration of siRNA in mice leads to robust 80-90% knockdown of the target gene in liver, kidney, spleen, lung and pancreas 106. Importantly, most *in vivo* studies of siRNA focus on proof-of-principle therapeutic efficacy in the target organ and rarely address the issue of on-target side-effects. It is clearly important to develop specific organ-directed target delivery. Various ligands and conjugates have improved the specificity of distribution to target tissues. But these measures are partially effective at best and studies generally show liver, spleen or kidney uptake at levels similar to or even higher than the target tissue. For example, epidermal growth factor receptor (EGFR) ligand enables more specific targeting of non-small cell lung cancer xenografts 107; vascular cell adhesion molecule 1 (VCAM1) antibody allows targeting of inflamed endothelium 108, 109 and folic acid can aid targeting ovarian of ovarian cancer xenografts 110, 111. However, anti-VCAM1 targeted lipoplexes improved lung uptake from 20% to 50% ID/g, but concurrent liver and spleen uptake was 60-70% ID/g in both groups 108. Histopathological evidence of chronic inflammation was found in non-target organs after folic acid-targeted treatment of mice xenografts with siHuR 110. Serum markers of liver and kidney function and tissue histology are often used to indicate toxicity in non-target organs but direct measurement of target gene expression in non-target sites is seldom available. On-target side-effects have been reported in clinical trials as discussed in the next section.

### Context-dependent effects of miRNA mimics

The evidences of condition and cell/tissue-type specific miRNA effects are accumulating. Functional studies of miRNAs have focused on their roles in disease models. Nonetheless, studies which have examined the baseline phenotype of transgenic mice and that of control animals receiving miRNA treatment; and comparisons of adjacent healthy and diseased tissue in the affected organ, have offered fair comparisons of miRNA mimic effects between healthy and diseased states. *In vitro* evidence also attests to the safety of the mimics as anti-tumor therapies.

#### Healthy *vs.* diseased

Transgenic mice with cardiac-specific overexpression of miR-133a have normal baseline cardiac function. Under pressure overload, which usually lowers expression of miR-133a in the heart, over-expression of miR-133a protected the heart against myocardial fibrosis and modulated electrical repolarization 112. In our own experience, miR-221 mimics protect cardiomyocytes against MI through dual anti-apoptotic and anti-autophagic effects by targeting *P53*, *Bak1* and *Ddit4* in the infarct area. Meanwhile, miR-221 mimics do not affect the myocardium remote from the infarct, where the expression of these genes is not changed nor are cellular apoptosis and autophagy as assessed by levels of cleaved Caspase 3 and LC3 respectively. Therefore miR-221 mimics do not affect the healthy myocardium 113. MiR-29b mimics significantly reduced targets *Col1a1* and *Col3a1* mRNA levels and hydroxyproline content in the rodent model of bleomycin-induced pulmonary fibrosis but not in healthy controls 81. MiR-29b mimics do not affect target expression in liver, kidney, spleen, heart or lung in healthy animals. MiR-302 mimics targeting *Mob1b* strongly increased cell proliferation in post-myocardial infarction (MI) hearts as indicated by CCND1 staining, a cell cycle marker, but had far less effect in sham hearts (~30% of MI) 114. Importantly, miR-302 mimics exhibit no toxicity in liver, lung or intestine, although uptake is more than 10-fold higher at these sites compared to the heart. Both studies showed wide distribution of mimics after systemic delivery but no side effects in healthy organs. MiR-664a significantly induced cell apoptosis in 3 human breast cancer cell lines with P53 mutation but not in P53 wild type cell lines 115. The context-dependent effects of miRNA mimics in health versus disease offer an important potential safety advantage.

The mechanisms underlying context-dependent miRNA regulation are not understood. Taking a more comprehensive bioinformatics approach, Erhard *et al.* analyzed four AGO2-PAR-CLIP (cross-linking immunoprecipitation) datasets from B cells at different developmental stages and disease conditions 116. They discovered widespread context-dependent miRNA-target mRNA interactions. Comparing the CLIP datasets with mRNA microarray, they found that the abundance of target mRNA could not explain their level of interaction with the miRNAs. Together the results support a stronger effect of miRNA in stressed or diseased conditions than in healthy homeostasis. This is an under-investigated area. Understanding of the underlying mechanisms, as further considered later in this review, would enhance the development of mimic therapeutics.

#### Cell/tissue-type specific

Cell/tissue-type specific miRNA expression has long been recognized, such as liver-specific miR-122, heart-specific miR-133 and miR-208, and so on. By analyzing 79 human tissues, targeted mRNA expressions are negatively correlated to tissue-specific miRNA expression 117, 118. In a MI mouse model treated with anti-miR-92, 4 distinct cardiac cell types: endothelial cells (EC), cardiac myocytes (CM), cardiac fibroblasts (cFB) and CD45+ hematopoietic cells, were isolated using specific markers 119. The upregulation of target genes, as assessed by RNAseq, clearly differed between cell types. EC were enriched in expression of autophagy regulatory genes whereas CM showed enhanced expression of metabolism-related genes. These results further support the principle of cell type-specific miRNA expression differentially influencing gene expression profiles in different cell types.

Both *in vitro* and *in vivo* evidence from individual miRNA studies supports the concept that miRNA functions are cell-type dependent. In vascular smooth muscle cells (VSMC) MiR-221/222 promoted proliferation, migration and were anti-apoptotic but had the opposite effects in EC 120. In balloon-injured rat carotid arteries, miR-221/222 overexpression increased neointimal formation and reduced re-endothelialization. Differential targeting on *P27, P57* and *c-Kit* may contribute to these findings. In our own experience, miR-221 mimic is anti-apoptotic through targeting of p53 in CM but this is not observed in cFB in a rat MI model 113. MiR-101a mimic induced apoptosis in cFB but not CM in a cardiac pressure overload rat model. MiR-101a mimic reduced cardiac fibrosis and improved cardiac function 121.

The mechanisms underlying cell type-dependent miRNA activity are critical yet under-investigated. Profiling gene regulation by a panel of miRNA mimics in 3 human cell lines and 5 tissue types, revealed ~10% of predicted targets presented 3'-UTR shortening. This variation dictates the presence or absence of miRNA binding sites on target mRNAs in different cell types 122. In addition to 3'- UTR shortening, isomiRs and RNA binding proteins may affect miRNA targeting. These issues will be discussed in later sections.

### Synergized multi-target miRNA effects

Seed region-mediated miRNA-target mRNA binding endows miRNA with an important attribute, the ability to bind to dozens or even hundreds of gene targets with relatively weak gene suppression of about 30%-60% 123-125. However, the aggregate of multiple mild changes adding up to network effects potentially make miRNA a powerful therapeutic agent. *H-Ras* and *HMGA2* are two oncogenes which mediate cancer cell proliferation and differentiation respectively 126, 127. Let-7 mimics downregulate both genes and reduce cancer cell proliferation and differentiation *in vitro* and reduce tumor volume by more than 70% *in vivo* 128. Knockdown of *H-Ras* or *HMGA2* by siRNA could only recapitulate the let-7 effects on self-renewal or differentiation respectively despite stronger downregulation of each gene individually. A double knockdown of *H-Ras* and *HMGA2* was not tested in this study. Let-7's tumor suppressor activities involve the suppression of many targets within multiple cellular pathways, *e.g.* cell cycle related genes including *CCND2*,* CDK6* and *CDC25*, cell polarization and migration related genes, *IGFBP1* and *IGFBP2* and transcriptional factors *E2F6, SOX9, YAP1* and others 129. It is impossible to simultaneously target so many coordinated genes with siRNAs. Similarly, in a study of anti-angiogenesis, miR-135a-3p directly targets huntingtin-interacting protein 1 (*HIP1*) and inhibits endothelial cell migration. SiRNA knock down of *HIP1* could only partially recapitulate this effect (<50% compared to mimics) even though the siRNA clearly knocked down *HIP1* itself more effectively than the mimics (85% vs. 30%) 130. Interestingly, combined siRNA and miRNA mimics treatment did not further enhance the anti-migration effect. This suggests that miRNA knockdown of any individual targets is generally mild-moderate but miRNA-induced mild knockdown of multiple targets induces salutary phenotypic changes.

### Synergized multi-miRNA effects

A single gene may be regulated by multiple miRNAs which can have additive effects and significant biological effects. Synergism is commonly observed in miRNA clusters. About 40% of total human miRNA loci are adjacent (≤ 10kbp), forming miRNA clusters. The expression of clustered miRNAs show strong positive intra-cluster correlation 131. MiRNAs expressed in the same cluster often have related biological functions 132. The signature of cancer is loss of normal control of cell proliferation by cyclin-dependent kinase inhibitors *(CDIs), e.g. P21Cip1, p27Kip1, p57Kip2,* functioning as brakes on the cell cycle. These important check-points are regulated by four clusters of miRNAs including the miR-17 cluster (miR-17, miR-20a, miR-92), miR-221 cluster (miR-221 and miR-222), miR-106b cluster (miR-106b, miR-93 and miR-25) and the miR-106a cluster (miR-106a, miR-20b, miR-92-2 and miR-363) 133. The dysregulation of these miRNAs and associated down-regulation of CDIs are reported in variety of cancers 132. A miRNA's cumulative integrated effects are not necessarily limited to its cluster, but can extend to sequence-based targeting, correlation-based expression regulation 134. For example, miR-34a and miR-15/16 are unrelated miRNAs, independently discovered as cancer therapeutic targets. Co-targeting with a mixture of miR-34a and miR-15/16 mimics showed synergy in inhibition of cancer cell lines. Interestingly, the synergistic effects were specific to cell cycle arrest but not cell apoptosis 135. The targeting of multiple miRNAs with synergistic effects may be an effective therapeutic approach worthy of further investigation and development.

## Unique regulations of miRNA function

**Figure 2:**
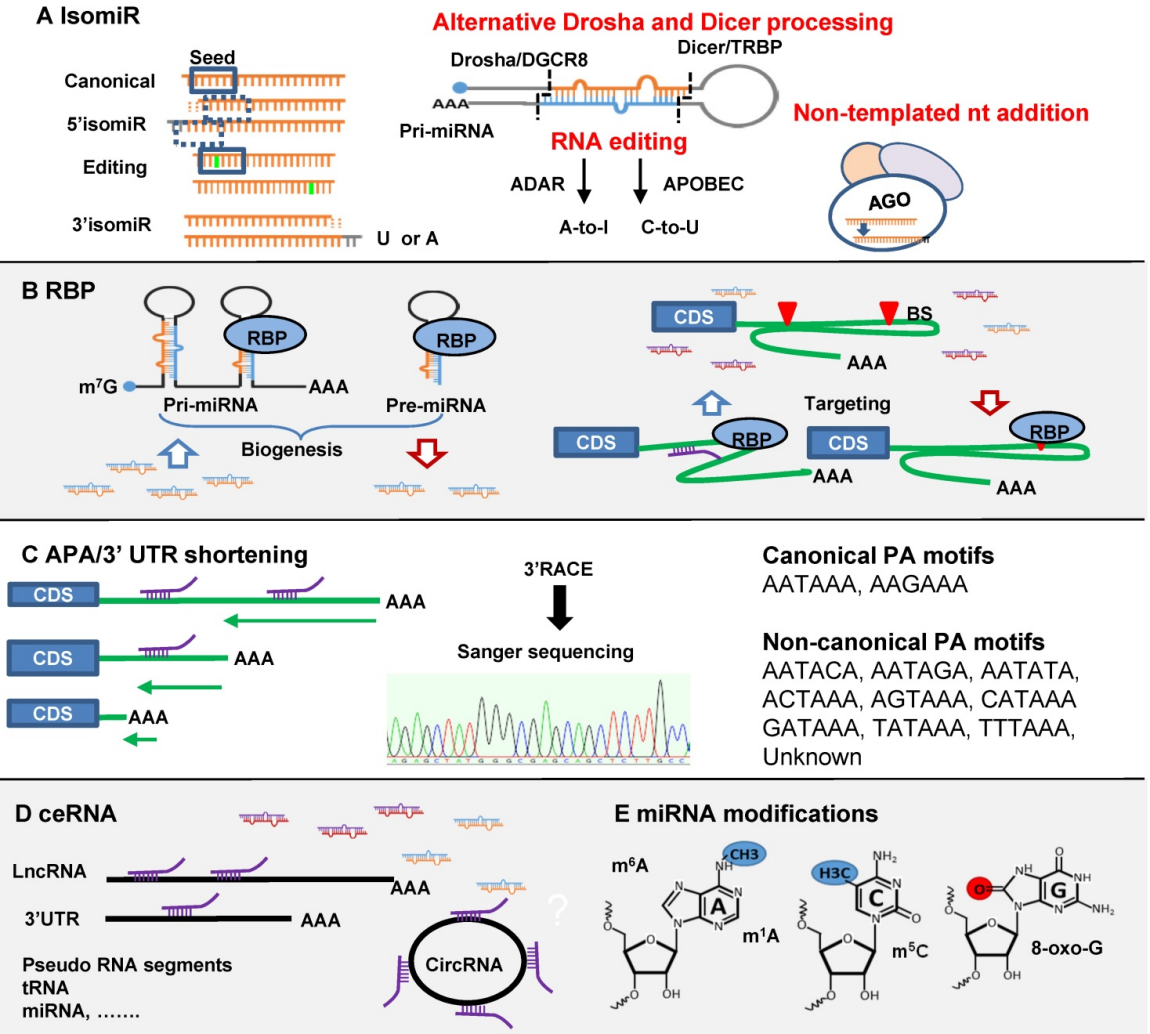
** Regulations of miRNA-mRNA interaction. (A)** IsomiRs are generated from three different mechanisms of alternative Drosha and Dicer processing, RNA editing and non-templated nt addition (NTA). MiRNA isomiR formation may affect seed region, miRNA loading and turnover. **(B)** RNA binding protein (RBP) (1) increases or decreases miRNA biogenesis through targeting pri- and pre-miRNA; (2) affects miRNA targeting positively or negatively through binding to mRNA 3'-UTR. **(C)** Alternative polyadenylation (APA)/3'-UTR shortening may cause the loss of miRNA binding sites on mRNA 3'-UTR. **(D)** The competing endogenous RNA (ceRNA) functions as a miRNA sponge to regulate miRNA function. ceRNA includes circRNA, lncRNA, 3'-UTR tail, pseudo RNA etc. **(E)** miRNA modifications including 5-methylcytosine (m^5^C), N^6^-methyladenosine (m^6^A), N^1^-methyladenosine (m^1^A), and reactive oxygen species (oxo-G).

Compared to siRNA interference, miRNA mediated gene regulation seems more complex. It is cell type and condition-dependent and affected by multiple regulatory factors. In other words, miRNA function is subject to multi-layer regulations. The underlying mechanisms are largely unknown. Again, in-depth understanding these mechanisms will facilitate the development of miRNA mimic therapeutics. Different mechanisms are summarized in Figure 2.

### IsomiRs

Small RNA deep sequencing has revealed that many miRNAs harbor slight sequence variations at different positions compared to their canonical versions 136. These isomiRs are common, and the profiles are cell-type specific and context dependent, cancer cells vs. healthy controls 137-139. A study in human brain samples revealed 80-90% miRNAs have isomiRs, predominantly 3' trimmings or additions 140. Sequence-oriented isomiR annotation (CASMIR) for unbiased identification of global isomiRs indicates that specific isomiRs are often more abundant than their canonical forms 139. IsomiR dysregulation has been reported in cancer, Parkinson's Disease, Alzheimer's Disease and Huntington's Disease 138, 141-143. Therefore, isomer profiling may have biomarker potential. The functional significance of isomiRs is not yet well understood. Many studies indicate 5' isomiRs function through altered regulation of gene targets 137. miR-411 5' isomiR with an additional adenosine is over 5-fold more abundant than canonical form in primary human vascular cells and differentially regulated under ischemia. Target prediction indicated 642 potential targets for miR-411 and 1249 for the 5' isomiR with 269 overlapping targets. Selected targets were validated in 3'UTR gene luciferase reporter assays: *TGF-β2* (miR-411), tissue factor F3 and *ANGPT1* (5'isomiR-411), *CDH2* and *CDH6* (shared). The 5' isomiR negatively regulated cell migration whilst miR-411 had no such effect 144. Further, 5' isomiR may affect AGO loading as U favors AGO1 but A favors AGO2 and AGO4 145. Functional alteration by 3'-isomiR has been less often reported but is generally believed to affect miRNA stability and turnover 45, 146. MiR isomiR length variation may affect miR function as well. For example, miR-222 is anti-apoptotic but isoforms with 3'-extension show pro-apoptotic activity through inhibition of the *PI3K-AKT* pathway 147.

Three mechanisms are proposed to explain isomiR biogenesis (Figure 2A). (1) Alternative Drosha and Dicer processing: a study of 5' isomiR-441 showed that adenosine deaminase acting on RNA (ADAR1 and ADAR2) affected the activities of DROSHA and DICER and facilitated 5' and 3' isomiR production 144. DROSHA processing generated 5' isomiRs of miR-142 were first reported in mouse T cells 148. Dicer modulated by TAR RNA-binding protein (TARBP) produce isoforms due to site variation cleavage 149. (2) RNA editing: a post-transcriptional modification process observed in mRNA and non-coding RNA such as miRNA 150. The editing predominantly happens at the pri-miRNA stage, but in principle it could happen in pre-miRNA. There are two canonical forms of editing, adenosine to inosine (A-to-I) and cytosine to uracil (C-to-U) mediated by ADAR and APOBEC1 (apolipoprotein B mRNA editing enzyme, catalytic polypeptide-like 1) respectively 151, 152. The first A-to-I editing was reported in pre-mir-22 in human and mouse 150. A comprehensive analysis of 767 million human sequencing reads identified 22688 RNA editing events spread out in coding and non-coding genes. Among them 44 editing sites were found in miRNAs 153. MiRNA editing is condition- and tissue-dependent and leads to the interference with processing by Drosha or Dicer and seed region alteration-induced target changes 150, 154-156. (3) Non-templated nt addition (NTA): the majority of miRNA isomiRs are 3' additions of tailing and trimming generated through NTA. This additional nucleotide tail of adenylation (A) or uridylation (U) cannot be matched to the precursor sequences 157, 158. Among them, U-addition is more frequent than A-addition; 56% vs. 12% of total 3'-additions 139. In a global analysis of miRNA metabolism, 5-ethynyluridine (5EU) metabolic labelling and biotin pulldown of nascent miRNAs followed by deep sequencing revealed that after loading into AGO, mature miRNAs are subject to 3' additions 45. This is the main process, generating isomiRs. U-tailed isomiRs are produced at the fastest rate, then trimming and A-tailing. 3' addition greatly affects miRNA turnover 45. However, U-addition may inhibit miRNA activity as evidenced of miR-26b in a human adenocarcinoma cells 159. At least three exoribonucleases and seven nucleotidyl transferases are reported as implicated in this process 146. NTA could generate 3' isomiRs from miRNA mimics as well. Small RNA sequencing data from Hela cells transfected with miR-17-92 family mimics revealed frequent modification of the guide strand sequences within 6 hr after transfection 160. In rat post-infarction heart we found significantly increased miR-221 3' isomiRs after treatment with miR-221 mimics. Fold changes in isomiRs were similar to concurrent increases in the canonical form (unpublished data). This is a largely untouched area in miRNA mimic therapeutics which may cause *in vivo* alteration of mimic function and potentially produce unexpected effects.

The discovery and quantification of isomiRs is heavily dependent on small RNA deep sequencing. Technical hurdles remain in this area. The widely used stem-loop qPCR for miRNA quantification cannot distinguish small differences between isoforms 161, 162. Novel methods such as dumbbell qPCR and two-tailed RT-qPCR have been developed for reliable isomiR assessment 163, 164. These are not yet available as off-the-shelf commercial kits. Their distribution for functional validation of isomiRs are urgently needed to facilitate isomiR research.

### RNA-binding proteins (RBPs)

RBPs function as post-transcriptional regulators and play essential roles in RNA/miRNA biogenesis, transport, stabilization, and function 165. RBPs comprise a large family with diverse function. AGOs for miRNA binding in miRNA biogenesis and functional machineries. The RNases DROSHA and DICER may have such effects but are not discussed. Here we focus on the regulatory effects of RBPs affecting miRNA biogenesis and miRNA-target interactions (Figure 2B).

MiRNA biogenesis is precisely controlled at all steps. Like transcription factors, RBPs preferentially interact with gene promoters and affect their activity; *e.g.* TP53 regulates miR-34 family transcription 166, 167. Besides this, through interacting with pri-/pre-miRNAs RBPs can post-transcriptionally regulate mature miRNA expression level. Systematic discovery of RBP-miRNA interactions has been reported using either synthetic miRNA probes to capture RBPs followed by identification via mass spectrometry or specific antibodies to immunoprecipitate (IP) RBPs followed by sequencing of cross-linked miRNAs. Around 180 RBPs were identified as interacting specifically with 72 pre-miRNAs. Interactions with pri-miRNA were discovered as well, *e.g.* ZC3H10 with pri-miR-143 168. By analyzing publicly available eCLIP datasets, 126 RBPs were annotated to bind at 1,871 human pre-miRs and more than 146 RBPs interact with pri-miRNAs in HepG2 and K562 cells 169. Using RBP gain and loss of function coupled to bioinformatics analysis, RBP-induced post-transcriptional regulation increases or decreases miRNA expression level through regulation of miRNA processing and/or stability. The regulation is: (1) context-dependent as evidenced by distinct “interactome” profiles in different conditions and cell types; and (2) sequence-dependent, as a particular RBP binds to miRNAs with certain sequence motifs. RNA secondary structure and co-factors also play a role 168, 169.

RBP binding to target mRNA 3'-UTR can either facilitate or inhibit miRNA-target interactions by altering the accessibility of miRNA binding sites in a condition-dependent fashion. This process could affect miRNA mimics. Although it has not been systematically studied, effects of well-known RBPs on miRNA-target interactions have been documented. The AU-rich element binding protein (HuR), one of the best studied RBPs, binds to p53 3'-UTR and stabilizes it for translation in response to DNA damage 170. In response to stresses or mitogenic stimuli, HuR translocated from the nucleus to the cytoplasm and regulates miRNA-target interactions either positively or negatively 171-173. Binding of HuR to *c-Myc* 3'-UTR facilitates the targeting of let-7, while binding of HuR to *ERBB-2* 3'-UTR antagonized miR-331. These effects are completely or partially lost upon siRNA knockdown of HuR 174, 175. Pumilio (PUM) is another well-known and highly conserved RBP family. PUM binding motifs are enriched near miRNA binding sites 176. Through inducing conformational change of mRNA structure, PUM1 mediates miR-221/222-induced targeting on cell cycle suppressor P27 and promotes cancer cell proliferation 177-179. The *p27* 3'-UTR sequence forms a hairpin structure blocking miR-221/222 binding sites in quiescent cancer cells. PUM1 is recruited to *p27* 3'-UTR and induces structural change exposing the miR-221/222 binding site 179. We observed that, due to lack of cardiac PUM1 expression, miR-221 does not downregulate *p27* in the heart 180. Accordingly, miR-221 mimic improved cardiac function after MI, without inducing cell proliferation 113. We and others have shown that miR-221 mimic is a promising cardioprotective therapeutic with anti-fibrotic effects 113. The requirement of PUM1 for miR-221 induced cell proliferation mitigates the risk of oncogenesis in cardiac miR-221 mimic applications. Similarly PUM1 binding is important for gene silencing by miR-410 181. These examples suggest important roles for RBPs as miRNA co-effectors or antagonists. Together they form complex post-transcriptional gene regulatory networks and feedback loops.

### Alternative polyadenylation (APA)

It is also termed as 3'-UTR shortening. Binding of miRNAs to the 3'-UTR of the target mRNA was observed in the earliest reports of the miRNAs lin-4 and let-7 2, 182 and later confirmed as a widespread phenomenon by systematic discovery of conserved miRNA binding motifs in 3'-UTR 183, 184. 3'-UTR binding remains the dominant target prediction algorithm. Therefore, 3'-UTR shortening can lead to loss of the binding site and switch off miRNA- target regulation (Figure 2C).

Polyadenylation (PA) is an essential step in mRNA maturation. The nascent mRNA is cleaved 10-30 nt after the PA signal motif and an untemplated poly(A) tail is added 185. The most common PA motif, AAUAAA and AUUAAA, is usually located at the end of the 3'-UTR 185. More than ten single-base variants of the hexamer sequence, non-canonical PA motifs, have been identified in human genes that can also direct APA 186, 187. APA is regulated by the cleavage factor protein complexes (CFIm and CFIIm) 188. Cleavage and polyadenylation specificity factor subunit 5 (CPSF5 or CFIm25), encoded by *NUDT21* gene, is one of the best studied components. On survey of more than 10,000 genes, 54% of human and 32% of mouse genes have more than one poly(A) site, with conservation of APA patterns between human and mouse 189. Widespread 3'-UTR shortening by APA has been shown to contribute to oncogenesis, metastasis and is associated with poor outcome in cancer 190-193. More recent reports revealed NUDT21 dysregulation and 3'-UTR shortening of TGF-β-regulated genes as mechanisms contributing to fibrosis 194-196, cardiomyocyte hypertrophy 197, cell stress 198, mTORC1 signaling 199 and immune responses 200. APA is also a physiological mechanism involved in normal development and differentiation 201.

Loss of miRNA effects as a result of 3'-UTR shortening has been repeatedly documented. In skeletal muscle stem cells, *Pax3* transcripts were subject to APA. The short and long forms of *Pax3* mRNA were differentially regulated by miR-206, thus resulting in varying levels of PAX3 protein to direct cell differentiation and muscle function 202. Cardiac ischemic preconditioning induced APA of *HSP70.3* to generate a shortened isoform that lost the miR-378* binding site. Increased levels of HSP70.3 protected cardiomyocytes against injury 203. Interestingly, NUDT21 knockdown induced glutaminase APA, generating a short isoform which retained the binding site for miR-23. As a result, miR-23 mimics suppressed the short isoform, as assessed by luciferase reporter assay, much more powerfully than the full length 3'-UTR 204. The mechanisms are not clear but might be due to reduced mRNA secondary structure and/or faster deadenylation 205, 206. As 3'-UTR contains other regulatory elements for RBP, 3'-UTR shortening could affect RBP regulation of miRNA targeting 198.

3'-UTR shortening-mediated escape from miRNA regulation or enhanced gene silencing by miRNA may affect the efficacy of miRNA therapeutics. It may form a layer of regulation partly explaining the context-dependent nature of miRNA regulatory function. Indeed, wide-spread cell-type specific APA patterns have been discovered through analysis of single cell sequencing data 207. Better understanding of the 3'-UTR landscape in disease is required for the design and testing of miRNA mimic treatments.

### The competing endogenous RNA (ceRNA)

Endogenous circular RNA (circRNA), long non-coding RNA (lncRNA), pseudogene transcripts and other RNAs can act as natural miRNA sponges through their miRNA complementary binding or promote degradation of miRNAs 208, 209 (Figure 2D). The profiling of circRNA expression from 20 human tissues has demonstrated that circRNA expression is highly tissue-specific and an important clinical biomarker 210. The functions of circRNA are complicated and not yet fully understood. However, there is plentiful evidence of circRNA being a sponge sequestering miRNAs. For example, the circRNA sponge for miR-7 (ciRS-7, also called CDR1as), highly expressed in human and mouse brain, contains more than 70 miRNA binding sites 211. Overexpression of CDR1as potently inhibits miR-7 and impairs brain development 212. Similarly, lncRNA, by definition as non-translatable RNA more than 200 nt in length, is a large family. However, bi-functional lncRNAs, protein coding and non-coding, have also been reported. In 2007 a study reported that only 1/5 transcripts are protein-coding RNAs 213. Now there are 270,044 lncRNA transcripts in human with complicated predicted miRNA interactions (http://bigd.big.ac.cn/lncbook/index) 214. The expression is cell type dependent and functionally associated with diseases 215. One of the earliest studies demonstrated that linc-MD1, a muscle-specific lncRNA, regulates myoblast differentiation through sponging miR-133 and miR-135. The dysregulation of linc-MD1 plays an important role in Duchenne's muscular dystrophy 216. LncRNA function as a miRNA sponge has obvious potential to influence miRNA mimic therapeutics. More complicated lncRNA functions are reviewed elsewhere 217, 218.

A recent study is worth mentioning here, as providing a potentially novel source of endogenous miRNA sponges. Through transcriptome-wide analysis of 5' capped and uncapped mRNA sequences in human cell lines, thousands of stable uncapped 3'-UTR tail-end fragments were discovered, as cleavage products of APA 219. It is generally believed that uncapped mRNA sequences are unstable and rapidly degraded 220. However, this study showed for the first time, that mRNA segments without 5' caps can remain stable within cells. The 3'-UTR fragments carry miRNA binding sites but are separated from the coding sequences. Theoretically they might sequester miRNAs like miRNA sponges. This is an untouched area worthy of study. By analyzing 108 published data set from CLIP-Seq (HITS-CLIP, PAR-CLIP, iCLIP, CLASH) and degradome sequencing, Li et al. established starBase v2.0, a database for decoding miRNA-mRNA, miRNA-lncRNA, miRNA-sncRNA, miRNA-circRNA, miRNA-pseudogene, protein-lncRNA, protein-ncRNA, protein-mRNA interactions and ceRNA networks 221.

### MiRNA modifications

RNA modifications which involve chemical modification of the base nucleotides have been extensively studied in mRNA. Over one hundred types of chemical modifications have been discovered, including the well-known 5' methylated guanosine cap (m^7^G) and the highly prevalent N^6^-methyladenosine (m^6^A). These chemical modifications alter mRNA function or stability 222. MiRNA modifications remain largely unexplored. A few studies have shown that endogenous miRNAs are also subject to natural chemical modifications (Figure 2 E). M^6^A marks have been found enriched in pri-miRNAs. This modification is critical in their recognition and processing into pre-miRNA by Drosha/DGCR8 223. Methylated mature miRNAs (5mC, m6A, m1A) have been detected in gastrointestinal cancer cells. These miRNAs showed significantly higher methylation levels in pancreatic and colorectal cancer vs normal control tissues, along with upregulation of RNA methyltransferases 224. MiR-184 was shown to be oxidized by reactive oxygen species in H_2_O_2_ treated H9c2 cells. This modification led to gain-of-function effects upon 2 new targets, *Bcl-xL* and *Bcl-w,* and induction of apoptosis. The same study also detected changes in gene regulation patterns by 2 more miRNAs upon oxidation 225. These studies strongly indicate the existence and likely importance of miRNA modifications that warrant further investigation.

## MiRNA function assessments in need: prediction and validation

The existing miRNA target prediction algorithms are primarily based on the complementary binding of miRNA seed region to mRNA 3'-UTR BS, which is known as canonical targeting. The common used methods for prediction are seed match, thermodynamic stability, conservation between species and target site accessibility. The platform Tools4miRs (https://tools4mirs.org) provide more than 160 tools for miRNA analysis under categories: Known miRNA identification, isomiRs identification, Novel miRNA/Precursor analysis, Differential expression analysis, Target prediction, Target functional analysis and miRNA-SNP analysis.

### Non-canonical miRNA target interactions

Non-canonical miRNA-target interactions implicate complementary binding beyond 3'-UTR BSs of mRNA, such as coding region, 5'-UTR and gene promoter, and/or beyond seed region of miRNA, such as bulges, G:U wobbles, mismatch or “seedless” binding 226, 227. The non-canonical miRNA-mRNA interactions have been well documented and are summarized in Table 2. The first discovered miRNA lin-4 target *Lin-14* contains *7* 3'-UTR BSs and 4 of them bear bulged C 228. A bulged A seed sequence was reported on the let-7/*Lin-41* duplex 229. MiR-24 binds seedlessly to 7 target mRNAs of E2F2, MYC, AURKB, CCNA2, CDC2, CDK4 and FEN1, to inhibit proliferation of human leukemia cells 230. MiR-20 seed region mutation studies demonstrated strong dependence of the miRNA 3'-end sequence targeting *DAPK3* CDS 231. MiRNA target genes in their coding region, *e.g.* miRNA-296-Nanog, miR-470-Nanog, miR-470-Oct4 and miR-134-Sox2 play an important role in mouse embryonic stem cell differentiation 232. We have discovered individual non-canonical miRNA-target pairs such as miR-221 and p53, validated by 3'-UTR cloning with binding site mutagenesis and luciferase reporter assays, demonstrating functional significance through anti-apoptotic signaling *in vitro* and *in vivo* 113.

**Figure 3:**
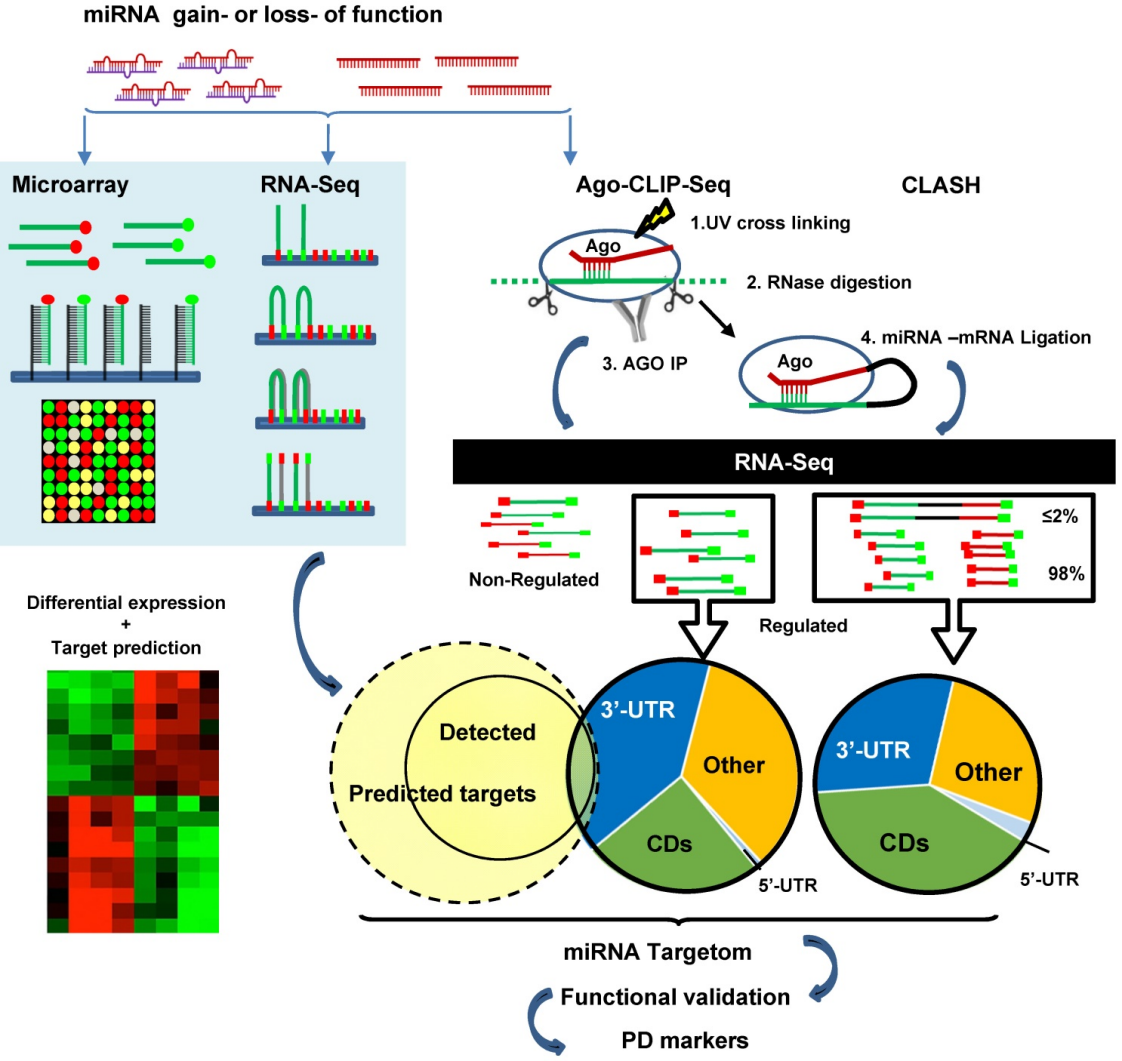
** MiRNA targetome analysis.** Traditional approach (left): gain- or loss-function manipulations followed by mRNA sequencing or microarray. MiRNA-induced gene regulation is determined by comparing the mRNA dysregulation data set with the target prediction data set. Advanced approach (right): high-throughput sequencing of RNAs isolated by crosslinking immunoprecipitation of Ago (HITS-CLIP or AGO-CLIP). The approach identifies cross-linked AGO and RNA with UV, RNase treatment to digest RNA into ~50-100 nt fragments, AGO immunoprecipitation, AGO-binding RNA isolation and recovery, RNA sequencing, and bioinformatics analysis to infer miRNA-mRNA interactions. More advanced technologies are cross-linking, ligation and sequencing of hybrids (CLASH, also known as AGO-CLIP-Hybrids-Seq), a procedure with an additional step for inter-molecular ligation of RNAs 5' to 3' to form a hybrid or chimeric. As this procedure detects AGO-loaded mRNAs, non-canonical targets can be discovered.

The above studies rely heavily on cloning followed by luciferase reporter assay validation of individual miRNA paired with selected mRNA. High-throughput screening makes it possible to discover the full spectra of miRNA-target binding, revealing the prevalence of non-canonical interactions. High-throughput sequencing of RNAs isolated by crosslinking immunoprecipitation (HITS-CLIP, also known as CLIP-Seq) was first developed to identify neuron-specific RNA-binding protein 233, 234. Subsequently, HITS-CLIP of AGO (AGO-CLIP) has been applied to study miRNA targets. The procedure includes crosslinking AGO and RNA with UV, RNase treatment to digest RNA into ~50-100 nt fragments, AGO IP, AGO-binding RNA isolation and recovery, RNA sequencing, and bioinformatics analysis to infer miRNA-mRNA interactions 235, 236. AGO-CLIP is also known as differential HITS-CLIP (dCLIP) as it is often combined with miRNA gain- or loss-of-function studies to select specific miRNA regulated genes (Figure 3) 237. It is also known as the “RISCome” as RNAs are RISC enriched. To identify miR-133a targetom in the heart, mouse hearts with miR-133a overexpression were compared with wild type hearts. A total of 2149 RISC-enriched mRNAs were detected. Among them 209 are hyper-enriched miR-133 targets (targets significantly downregulated by miR-133) with 195 non-predicted, raising the possibility of non-canonical 3'-UTR binding 238. Similarly, to determine miR-155 targets in mouse primary T cells, T cells from wild-type and miR-155 knockout mice were studied. dCLIP revealed 40% non-canonical binding sites, including 5'-UTR, coding regions and the introns of genes, of total miR-155-target interactions. Most non-canonical targets were demonstrated to be regulated by miR-155 as assessed by luciferase reporter assay. However, the gene suppression effects of non-canonical targeting alone are much weaker than in canonical targeting 237. Other Ago-CLIP based studies gave estimates of the prevalence of non-canonical binding sites varying from 15-80% of total interactions 239.

Compared to traditional methods of RNA sequencing, AGO-CLIP directly assesses AGO-bound RNAs. However, the pairing between miRNA and mRNA sequences are computationally inferred rather than experimentally proven. To address this issue, miRNA-mRNA duplexes may be directly captured by crosslinking, ligation and sequencing of hybrids (CLASH, also known as AGO-CLIP-Hybrids-Seq). The major difference compared to CLIP is an additional step for inter-molecular ligation of RNAs 5' to 3' to form a hybrid or chimeric 240. Using this method, Helwak *et al.* obtained a large dataset of 18,500 human miRNA-mRNA interactions, revealing a high frequency of non-canonical miRNA-mRNA targeting 241. Although miRNA seed region targeting accounts for more than half of the interactions, 60% of them contain bulged, mismatched, G-U pairing or additional non-seed region pairing. There are substantial numbers of miRNA interactions with all regions of mRNAs. Notably, the proportion of miRNA pairing with mRNA at regions of 5'-UTR, CDS and 3'-UTR are miRNA-specific, *e.g.* miR-100 are 4%, 23% and 73% vs. miR-149 are 8%, 72% and 19% of three regions respectively 241. Overall miRNA BSs in 5'-UTR are rare, but BSs in the CDS appear equally or more frequently than in the 3'-UTR, comprising 40-60% of all AGO-bound mRNAs based on 3 different studies 235, 241, 242. BSs in CDS are conserved across species 243, 244. MiR-30 showed very similar inhibition effects between plasmid of 3'-UTR with 2 BSs and plasmid of extended opening reading frame (ORF) with 2 BSs by nucleotide insertion to abolish stop codon in NIH3T3 cells and *in vivo* overexpression of these plasmids 245. However, the function and mechanism of CDS targeting is not clear. By analysis of ribosome protected fragment sequencing and mRNA profiling, it is shown that sites located in the CDS are most potent in inhibiting translation versus canonical 3'-UTR binding which is more efficient in mRNA degradation. 246. CLASH studies also identified a substantial number of “seedless” interactions (16% of the total) in which miRNAs target mRNAs via their 3'region 241.

### MiRNA targetome

The collection of specific regulated targets through which a miRNA exerts its effects. It is challenging to identify complete targetomes in a context and cell-type dependent manner. This information is pivotal to the development of miRNA therapeutics. It involves *in silico* prediction and high-throughput validation technology. To date, the most commonly used prediction algorithms are based on the seed-pairing (*e.g.* TargetScan). As discussed above, non-canonical miRNA targeting has been well documented. By analyzing published AGO-CLIP-seq based data, Grosswendt *et al.* reported an additional ~13,000 miRNA-mRNA interactions 247. Full revelation of miRNA targetomes is not possible by traditional experimental studies which only allow validation of one or a few miRNA targets at once. A quick PubMed search show ~0.1% miRNA targetome studies over all miRNA related studies. There is a need for genome-wide studies of miRNA targetomes especially in human pathologies. Despite numerous studies indicating the pivotal role of miRNA in cardiovascular diseases, only two AGO-CLIP-seq studies have been reported to date 112, 248.

Sequence and IP-based technologies require refinement of both the experimental procedures and analysis of the data produced. From AGO1 CLASH, only 2% of reads are hybrids composed of mature miRNA ligated to a target RNA. The other 98% of the reads are single RNA, i.e. mRNA or miRNA 241. Efforts have been devoted to improve the efficiency of capture miRNA targets, for example photoactivatable ribonucleoside-enhanced crosslinking and immunoprecipitation (PAR-CLIP) and individual nucleotide resolution CLIP (iCLIP) have been developed for better resolution 242, 249. The comparison and applications of these technologies are well reviewed 236, 250. There is also a great need to improve computational CLIP-seq data analysis and/or algorithms and establish new databases 236. The understanding of miRNA targetomes will deepen with development of and further experience with these technologies.

### Pharmacodynamic markers (PD)

The assessment of RNAi-specific biological activity by PD markers is important in guiding and evaluating the effectiveness of treatment. They provide surrogates for functional improvement or survival benefit. PD may include the proof of mechanism, i.e., intended targets, and proof of concept, i.e. the desired molecular effect. In distinction from safety and efficacy biomarkers, PDs allow monitoring of molecular responses to a therapy. For siRNA drugs, the degree of target mRNA knockdown is the gold standard PD marker. Silencing of the liver specific gene *Ttr* by *Ttr*-siRNA in the rat can be confirmed by significant reductions of Ttr protein in liver and serum 251. A similar method has been successfully used in Givosiran treatment in which downregulation of ALAS1 can be monitored in serum and even in urine samples 252, 253. Besides TTR, many tissue specific gene products from liver, muscle, leukocytes, kidney and so on can be measured in serum in rats, cynomolgus monkeys and even in humans 251. However, target proteins are not always secreted into the circulation. For miRNA mimics, due to their multi-targeting properties and subtler mRNA downregulation, the development of a reliable panel of PD markers is challenging. The currently limited understanding of the miRNA targetome adds more uncertainties. Reliable PD markers in accessible samples remain an unmet need in many instances. Through examining the existing miRNA clinical trials and animal studies, some principles can be defined.

Trials of Remlarsen mimic treatment for skin fibrosis have incorporated attempts to discover and validate PD markers for miR-29b 254. In a mouse model, a total of 228 genes were significantly regulated by mimics and anti-miR in opposite directions. A panel of 24 genes, including 5 direct targets and 19 functional indications of ECM component/receptor, cell proliferation/differentiation and Notch/Wnt signaling factors, were selected as PD markers. The panel showed a dose-dependent response to miR-29b mimic treatment in mouse, rat, and rabbit tissue biopsy *in vivo* and in human skin fibroblasts *in vitro*. Next, by cross-species analogy, 16 PD markers were selected for clinical (human) trials. MiR-29 mimics have also been shown to block pulmonary fibrosis in a mouse model and human lung fibroblasts 81, 255. In all of these studies, no serum markers have been explored. In MRX34 studies a set of the 9 most-changed directly targeted genes and downstream functional genes were selected as a PD marker panel. This set of genes was consistently downregulated in tumor biopsies by MRX34 treatment in 2 mouse xenograft models. In clinical trials gene regulation was interrogated in circulating white blood cells (WBC). Top changes of 5 target genes were identified by RNA sequencing. However, due to different sample types, these two PD marker panels (derived in mouse xenograft tumor biopsies and in human WBC) had only 2 overlapping genes 256, 257. However, this is a valuable attempt to use feasible alternative samples other than tissue biopsy.

Traditionally, PD markers have been based on analyzing the differential expression of individual molecules. The advancement of high-throughput technology and bioinformatics, network-based gene expression analysis, *e.g.* gene regulation, signaling network and protein-protein interaction analyses provide tools for multi-PD marker panels 258. Computational literature analysis to facilitate PD marker discovery is under development 259. By observing the steps taken and rules applied, miRNA therapeutic PD markers should be targetome-based. First, miRNA PD markers should include multiple direct gene targets, and downstream signaling network and functional genes. Secondly, ideally PD markers should be measured in target tissues. However, tissue biopsies are rarely available and more accessible sample types including blood and/or urine should be investigated for PD utility. Case by case, agent-specific investigations are required to identify and validate PD for each candidate miRNA mimics therapy.

## Clinical trials

### SiRNA clinical trials

There are more than 38 studies registered for clinical trials with three FDA approved and a few discontinued (https://clinicaltrials.gov) 9, 260, 261. Major studies could be summarized under three categories of local delivery based treatments for degenerative blindness, cancer and liver diseases (Table 3). We will not summarize all the trials but highlight current progress, identify residual limitations and discuss whether miRNA mimics could potentially fill the functional gaps in achieving the optimal RNAi-based therapeutic.

#### Local delivery-based siRNAs

Given the concerns of on-target side-effects, ophthalmic drugs comprising naked siRNAs administered locally clearly avoid these obstacles. Sylentis Pharmaceuticals has two drugs formulated in eye drop solutions in Phase II or III clinical trials. **SYL1001 (Tivanisiram),** 11.25 mg/ml one drop q.d. for 28 days, silences the vanilloid receptor 1 (TRPV1) to treat dry eye syndrome 262, 263. **SYL040012 (Bamosiran),** 0.375%-1.5% one drop q.d. or b.i.d. for 28 days, silences the β2-adrenengic receptor to treat glaucoma. No systemic adverse effects were observed at any time in pre-clinical and phase I trials 264, 265. Quark Pharmaceuticals focus on the treatment of oxidative stress and ischemic injury (http://quarkpharma.com). **QPI-1007,** 0.5-10.0 mg/kg once after surgery,** and PF-655,** 1.5 and 3.0 mg once in 30 days, are naked siRNAs delivered by intravitreal injection to treat blindness. Through targeting of CASP2 and DDTT4 (also called RTP801) respectively, these siRNAs inhibit the loss of retinal ganglion cells and neovascular age-related macular degeneration 266, 267. The company has also moved forward beyond local treatment. **QPI-1002** targeting p53 has been developed to treat acute kidney injury or delayed graft function following kidney transplantation 268.

#### Anti-cancer siRNAs

The challenges for non-hepatic siRNA therapy may be best appreciated by reviewing experience with **ALN-VSP**, an anti-tumor siRNA formulation developed by Alnylam Pharmaceuticals 269. Two siRNAs, delivered by lipid nanoparticles, target vascular endothelial growth factor-a (VEGFa) and kinesin spindle protein (KSP) to reduce tumor microvascular density and to induce tumor cell mitotic arrest, respectively 270. Tumor blood flow was substantially decreased but cell mitotic arrest, as assessed by inspection for unipolar mitotic spindles in tumor biopsies, was not detected. In preclinical studies in rat and monkey models, siRNAs not only accumulated in the tumor tissue but also liver and spleen which caused the on-target side-effects of hepatoxicity and spleen/lymphoid atrophy. Similar results were reported in patients with primary liver carcinoma or secondary liver metastasis in phase I trials. Abdominal CT scans showed an average of 37% reduction in splenic volume (n=23) and one patient developed fatal liver failure. Specific cleavage products of *VEGF* mRNA were detected in biopsies from both normal and malignant liver tissues. The splenic atrophy was probably due to siKSP 271. Due to potentially lethal on-target side-effects in healthy organs, development of this siRNA was discontinued after phase I.

A handful of further anti-cancer siRNA clinical trials have been carried out. **TKM-080301**, 0.3-0.75 mg/kg 3+3 dose-escalation study once a week,** and ATU027,** 0.253 mg/kg once or twice a week, from Arbutus Biophama Corp. which target polo-like kinase 1 (PLK1) and protein kinase N3 (PKN3) inhibits cancer cell proliferation and vascularization respectively 272, 273. Both drugs have entered Phase I clinical trials in patients with advanced solid tumors. Adverse on-target side-effects included falls in neutrophil and platelet counts in addition to liver and spleen toxicity 274. G12D is the most prevalent mutation of the K-RAS gene found in many types of cancers. **siGI2D-LODER,** 3 escalated-doses of 0.025, 0.75, 3.0 mg once a week, is the only siRNA drug with a sustained release formulation by Silenseed Ltd. Through pancreatic implantation, it successfully suppressed tumor progression with limitation of adverse effects 275, 276.

#### Liver-specific delivery modification

On-target side-effects cannot be eliminated through improved siRNA design, altered sequence or chemical modification 277. For successful clinical application, targeted organ delivery is critical for improving drug efficacy and limiting adverse effects in non-target organs. The development of liver-specific delivery is advanced. Asialoglycoprotein receptor 1 (ASPGR1) first discovered by Gilbert Ashwell and Anatol Morell in 1965, is a transmembrane protein predominantly expressed on the hepatocyte membrane 278. Specific binding of N-acetylgalactosamine (GalNAc) to ASPGR1 leads to rapid endocytosis. During the maturation of endosomes, the increase in acidification triggers the dissociation of GalNAc and ASPGR1. They are, respectively, degraded and recycled to the cell membrane 279. The nonclinical safety study tested the organ distribution of 6 Alnylam GalNAc-siRNA drugs in Sprague-Dawley rats and cynomolgus macaques 86. High doses of 30 and 300 mg/kg, 30 to 300 fold higher than clinical dosages, were subcutaneously administered once a week for three weeks. These siRNAs are exclusively restricted to hepatocytes. Therefore, to specifically knockdown disease-causing liver-expressed RNAs, GalNAc-siRNAs comprise an excellent strategy for avoiding non-hepatic side-effects. Nowadays multiple GalNAc-siRNA drugs are undergoing clinical trials. Among them Alnylam is the leading company with three drugs already FDA-approved for clinical use.

#### FDA approved GalNAc-siRNA drugs

Transthyretin (TTR) is predominantly synthesized and released from the liver as a homotetramer. TTR gene mutation causes TTR protein misfolding and aggregation in tissues, resulting in TTR amyloidosis which ultimately leads to organ failure and death 280, 281. **Patisiran**, GalNAc-TTR siRNA 0.3-1.0 mg/kg single dose, reduces hepatic TTR production by 87% 282. Mutation of aminolevulinic acid synthase 1 (ALAS1) causes the accumulation of neurotoxic intermediates of aminolevulinic acid (ALA) and porphobilinogen (PBG), resulting in the rare genetic disease acute hepatic porphyria 283, 284. **Givosiran (ALN-AS1),** 2.5-5.0 mg/kg once a month for 4 times, targeting ALAS1 in the liver, reduces ALA and PBG levels by more than 90% 252. **Inclisiran,** 300 mg SC injection every 6 months, GalNAc-PSCK9 siRNA, is recently approved by FDA for the treatment of hyperlipidemia which affects more than a third of the world's population. Binding of PCSK9 to low density lipoprotein LDL receptors leads to receptor degradation and therefore reduced cholesterol clearance 285. A single dose of Inclisiran knocks down PCSK9 by 74%. Through increased LDL receptor availability and LDL cholesterol uptake, it reduces serum LDL cholesterol levels by 50% over 84 days 286. Inclisiran is a milestone in RNAi therapeutics as it is applied beyond rare monogenic diseases.

#### Phase I or II clinical trial GalNAc-siRNA drugs

More RNAi drugs by Alnylam are currently in early to late phase of clinical trials. Fitusiran, Lumasiran, Cemdisiran, ALN-AATD2, ALN-HBVO2, and ALN-AGT to treat hemophilia, primary hyperoxaluria, complement-mediated diseases, a1 liver disease, hepatitis B virus infection and hypertension, respectively. Other companies are also focusing on GalNAc-siRNA development. Arrowhead produces ARO-HBV, two siRNAs targeting the X and S genes of Hepatitis-B and ARO-AAT, targeting mutated α1 antitrypsin. Dicerna Pharmaceuticals produces GalNAc-siRNAs to target three different gene mutations causing primary hyperoxaluria, a life threatening genetic disorder.

In summary, this is a fast moving field. Reliable liver hepatocyte-specific siRNA delivery has been secured. Unfortunately, highly efficient and organ-specific ligand-receptor pairing, as exemplified by the hepatic GalNAc-ASPGR system, is not the customary experience and such organ-specificity is very unlikely to be achieved for many other siRNAs in the near future. This severely limits the applications of siRNA therapeutics. Might miRNA mimics offer some unique therapeutic opportunities and advantages?

### Clinical trials of miRNA mimics

The development of miRNA mimics in RNAi therapeutics is less advanced than that of siRNA. Notably, Miravisen (Roche), an antimiR of miR-122 is on the market to treat hepatitis C virus infection (HCV) 287. MiR-122 is a liver specific miRNA which plays an important role in HCV propagation. Miravisen, by subcutaneous administration, induced a dose-dependent reduction of HCV load with no significant adverse effects 287. Although it is a naked single-stranded antisense DNA oligo, this success is an evidence of the therapeutic power of miRNA regulation. To date, only 3 miRNA mimics have entered clinical trials. Key features of trial design and outcomes are summarized in Table 3.

#### Remlarsen

The miR-29 family has three members; miR-29a, miR-29b and miR-29c. They are enriched in fibroblasts and downregulated in fibrotic diseases 288, 289. The multiple targets of miR-29 include several collagens, other extra-cellular matrix (ECM) genes and genes in the TGF-β1 signaling pathway. Remlarsen (Miragen Therapeutics), a naked, chemically modified miR-29b mimic is delivered locally to skin wounds for the treatment of keloid 254. Through anti-fibrotic effects, Remlarsen inhibits proliferative scar formation in incisional wounds 254. Intuitively, local topical delivery suggests relative safety. However, documentation on organ distribution and possible off-target effects is not available. A Phase II, double-blind, placebo-controlled study of Remlarsen, 5.3 mg of 6 doses in two weeks, to test its efficacy, safety, and tolerability in subjects with keloid scarring, is ongoing.

#### TargomiRs

The tumor suppressor of miR-15/16 cluster was discovered in B cell lymphoma due to alteration of their chromosomal region and later found to be downregulated in a wide range of tumors 290. MiR-16 mimics exert their anti-cancer effects via multi-targeting of cyclin dependent kinases and the VEGF pathway inhibiting tumor growth together with inhibition of *BCL2* which promotes apoptosis 291-293. TargomiRs is a tumor suppressor comprising miR-16 mimic packaged in nonliving bacteria minicells with anti-EGFR bispecific antibody labelling. Delivered intravenously it targets EGFR-expressing cancer cells resulting in uptake by endocytosis, intracellular degradation and subsequent drug release 294. The safety and distribution of EGFR-minicells drug delivery was tested in a human tumor xenograft mouse model. Two hours after injection, 30% and 40% of the drug was distributed in tumor and liver respectively. However, at 6 and 24 hours, the drugs were mainly retained in the tumor at concentrations of ~500 and ~200 μg/g tissue 294. TargomiRs dose-dependently inhibited cell proliferation in 4 human lung mesothelioma cell lines and almost completely blocked colony formation. However, colony formation of normal mesothelial MeT-5A cells was unaffected 80. In patients with malignant pleural mesothelioma, TargomiRs, 5 billion copies of mimics, was effective in stabilizing tumor growth in 16 out of 22 patients 295. EnGeneIC Ltd. Has announced a phase II trial. The potential application of TargomiR in combination with chemotherapy or immune checkpoint inhibitors is under consideration. Registered trial information is not yet available.

#### MRX34

miR-34a mimic tumor-suppressor. The miR-34 family members comprising miR-34a, miR-34b and miR-34c, are downregulated in various tumors 256 296. These miRNAs are downregulated by p53 in response to DNA damage. MiR-34a, the best studied family member, targets more than 30 oncogenes controlling cell cycle cyclin-dependent kinase 4 (*CDK4*); metastasis and epithelial-mesenchymal-transition (EMT) including *WNT/β-catenin, MAPK*, *Hedgehog, VEGF* and *c-MET*; apoptosis of *BCL2*; and immune check point PD1 ligand 1 (*PDL1*) 297, 298. MiR-34a significantly reduced proliferation of 5 human lung cancer cells lines by 15-70% but had no effect in primary human T cells, normal human skin or lung fibroblasts. Administered intravenously and locally, miR-34a mimics suppress the growth of lung cancer xenografts 299. Trials of MRX34, miR-34a mimics encapsulated in lipid nanoparticles, have been undertaken in hepatocellular carcinoma and other advanced solid tumors with hepatic metastases. It inhibited tumor growth in 19 out of 66 patients evaluated. Successful delivery of miR-34a was verified by *in situ* hybridization studies on patient liver biopsies 257. The MRX34 trial was terminated in 2016 due to immune-related serious adverse events (AE) 257. It is speculated this reflects the multi-target effects of miR-34a including the inhibition of the immune checkpoint genes *PDL1* and *LGR4* 300 301. The syndrome is similar to that observed with PDL1 inhibitor treatment 302 with targeting of *LGR4* increasing the expression of *TNFa, IL11, CXCL5* and *CCL2*.

These trials have both shown proof-of-principle of the therapeutic efficacy of mimics in humans and highlighted the difficulties that remain. The failure of MRX34 illustrates a spectrum of detrimental alongside beneficial multi-target effects of miRNA. Thorough understanding of miRNA effects under different conditions and in different cell types is critical to the development of mimic therapeutics.

## Summary

MiRNA mimics and siRNA are highly similar synthetic ~22 nt dsRNAs and function via the same cellular RNAi pathway. The development of siRNA drugs is advanced with several siRNA drugs now FDA-approved for clinical use or currently in late-stage clinical trials. The development of miRNA mimics therapeutics lags behind. Our review has focused on the major differences between miRNA mimics and siRNA and highlights the potential advantages and challenges of miRNA mimics in RNAi therapeutics.

SiRNA is designed to silence a single target gene and selected for optimal efficiency, usually >80% downregulation of the target gene. The miRNA-like effects via seed-pairing might induce off-target effects. Through sequence comparisons and evolving design rules, deliberate chemical modification, massive experimental library screens and preclinical testing, off-target effects can be mitigated. SiRNAs ubiquitously knock down their target gene in any organ to which they are distributed. Without constrained organ-specific delivery, they may cause severe on-target side-effects. To date, all siRNA drugs are categorized as local delivery, end-stage cancers and more advanced in targeting the liver. The exceptionally efficient ligand-receptor pairing exemplified by GalNAc and ASPGR in the liver has not been paralleled for other ligands in other organs. This severely limits the applications of siRNA therapeutics. On the other hand, most diseases involve multi-gene dysregulation. Manipulating one gene may not effectively treat such diseases. MiRNA mimics might offer some unique therapeutic opportunities.

MiRNAs are endogenous post-transcriptional gene regulators, conserved across species. The microRNA-induced suppression of each individual target gene is relatively mild, yet functional effects are powerful due to synergistic suppression of multiple targets in integrated cell signaling pathways. MiRNA mimics are dsRNAs which comprise endogenous miRNA (guide strand) with modified passenger strand. As evolutionally selected, miRNA activities are regulated in response to different conditions in order to sustain homeostasis. Regulatory mechanisms, including isomiRs, RBPs, 3'-UTR shortenings and ceRNA sponges have not been well understood. Due to this complicated regulatory networking system, miRNA mimic applications are challenging but may offer advantages. MiRNA mimics show context dependency and may display different functionality under healthy versus diseased conditions or in different cell types. These unique properties offer novel opportunities in designing specific therapies that overcome the current limitation in wide bio-distribution of systemic delivery and lack of efficient targeted delivery to extra-hepatic organs.

Finally, a reliable and accessible penal of PD markers is important to guide and evaluate RNAi treatment. For current siRNA drugs targeting the liver, expression of the target mRNA is the gold standard PD and the change in the relevant serum protein correlates well with changes in the liver. For miRNA mimics, due to their multi-targeting properties and subtler downregulation of target genes, expression of multiple genes should be tested including miRNA direct targets and indirect, downstream functional gene targets. In many cases, target gene products are not released into the serum and tissue biopsy samples are not available. Agent-specific panels of PD markers are required.

Both siRNA and miRNA mimics hold therapeutic promise. The development of miRNA mimic therapeutics requires further elucidation of the mechanisms governing miRNA regulation and function. MiRNA mimics are a unique class of potential RNA-based therapeutics that offer distinct advantages and opportunities compared to siRNAs.

## Figures and Tables

**Table 1:** Major resources of commercial miRNA mimics

Category Details	
**MISSION® microRNA Mimics**	
**Company**	Sigma-Aldrich
**Library**	miRBase v17 human miRNA
**Modification**	“design significantly reduces possible passenger strand off target effects”
**mirVana Mimics**	
**Company**	ThermoFisher
**Library**	miRBase v22 all species miRNA
**Modification**	“chemical modifications prevent sense (passenger) strand entry into RISC”
**miRCURY LNA miRNA Mimics**	
**Company**	Qiagen
**Library**	miRBase
**Modification**	“LNA-enhanced complimentary strands prevent any miRNA-like activity”
**Dharmacon^TM^ miRIDIAN^TM^ Mimics**	
**Company**	Dharmacon.horizondiscovery
**Library**	miRBase v 21 human, mouse, rat miRNA
**Modification**	“modified to prevent sense (passenger) strand uptake”
**MIRacle^TM^ miRNA Agomir**	
**Company**	AcceGen Biotechnology
**Library**	miRBase human, mouse, rat miRNA
**Modification**	Antisense (guide) strand modified with: 5' 2 phosphorothioates, 3' 4 phosphorothioates and cholesterol, full length with 2'-methoxy

**Table 2:**
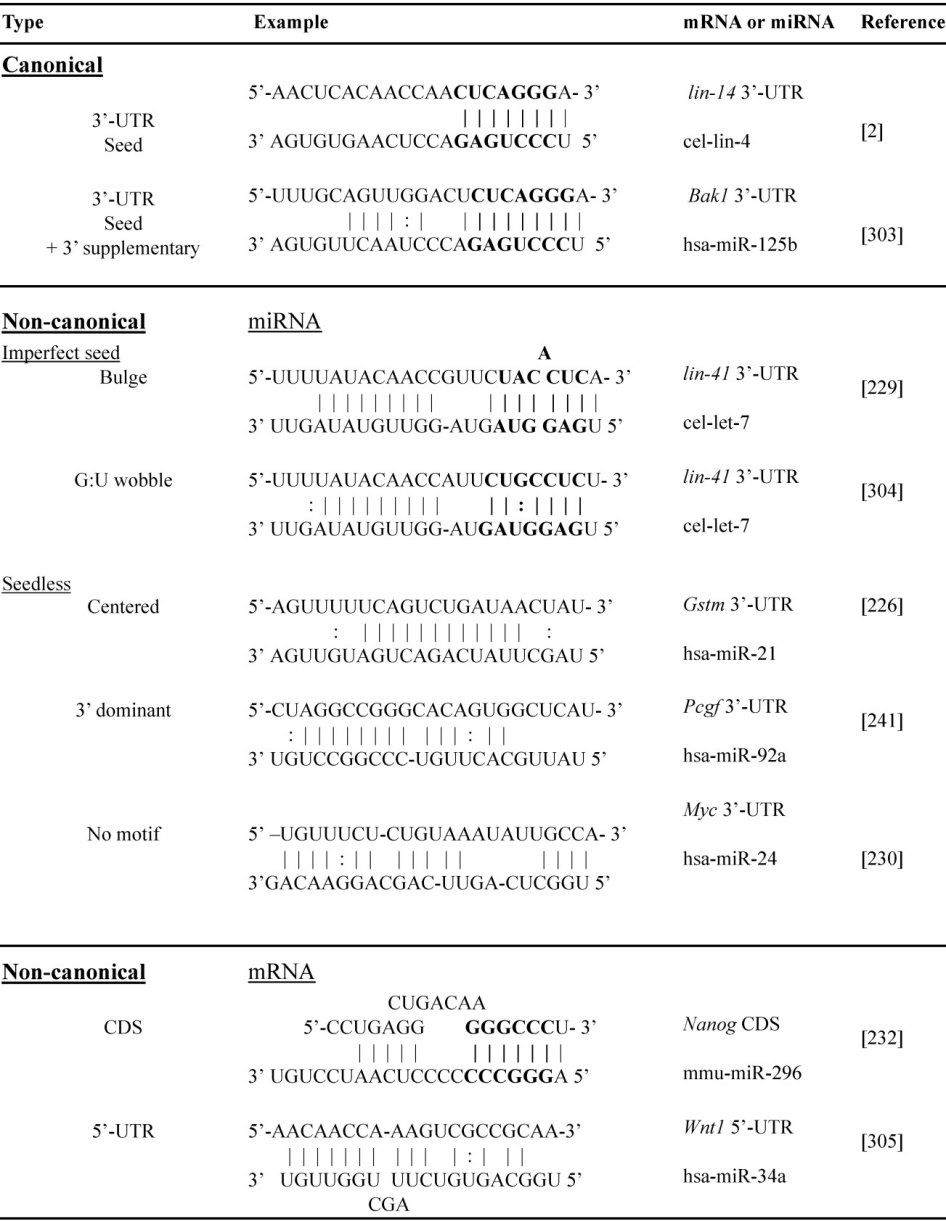
miRNA-mRNA canonical and non-canonical interactions

**Abbreviations**: 3'-UTR, 3'untranslated region; CDS, coding sequence; 5'-UTR, 5'untranslated region; cel, C. elegans; has, homo sapiens; mmu, mus musculus; Bak1, BCL2 antagonist/killer 1; Gstm, glutathione S-transferase mu; Pcgf, polycomb group RING finger protein; Myc, MYC proto-oncogene, bHLH transcription factor; Nanog, nanog homeobox; Wnt1, Wnt family member 1.

**Table 3:** Summary of representative siRNA and miRNA mimics clinical trials

Disease	Delivery	Company/Drug name	Target gene	Trial stage	Trial No.
**Naked siRNA local**					
Dry eye syndrome	Eye drop	Sylentis/SYL1001	TRPV1	Phase II	NCT03108664
Glaucoma	IVT	Sylentis/SYL040012	ADRB2	Phase III	NCT02250612
Blindness	IVT	Quark/QPI-1007	CASP2	Phase III	NCT02341560
Blindness	IVT	Quark/PF-655	RTP801	Phase II	NCT01445899
**Advanced Tumors**					
Solid tumors	i.v.	Arbutus/TKM-080301	PLK1	Phase I/II	NCT02191878
Solid tumors	i.v.	Arbutus/ATU027	PKN3	Phase I	NCT00938574
Pancreatic tumor	Implantation	Silenseed/siGI2D-LODER	G12D	Phase II	NCT01676259
**Lipid Nanoparticle siRNA**					
Acute kidney injury	i.v.	Quark/QPI-1002	p53	Phase III	NCT02610296
Solid tumors	i.v.	Alnylam/ALN-KSP	VEGFa + KSP	Discontinued	N/A
Transthyretin amyloidosis	i.v.	Alnylam/Patisiran	TTR	FDA approved	NCT01559077
**GalNAc-siRNA**					
Acute hepatic porphyria	s.c.	Alnylam/Givosiran	ALAS1	FDA approved	NCT02452372
Hypercholestrolemia	s.c.	Alnylam/Inclisiran	PCSK9	FDA approved	NCT03397121
Hemophilia A/B	s.c.	Alnylam/Fitusiran	Factor VIII	Phase III	NCT03549871
Hepatitis B virus infection	i.v.	Arrowheads/ARO-HBV	HBV- X and S	Phase II	NCT03365947
α1 liver disease	i.v.	Arrowheads/ARO-AAT	a1 antitrypsin	Phase II	NCT03945292
Hyperoxaluria	s.c.	Dicerna	LDH	Phase I	NCT03392896
**miRNA mimics**					
Keloid	Local	Mirna Ther./Remlarsen	miR-29b	Phase II	NCT03601052
Mesothelioma	i.v.	EnGeneIC/TargomiRs	miR-16	Phase II	NCT02369198
Liver cancer	i.v.	Miragen Ther./MRX34	miR-34a	Discontinued	N/A

**Abbreviations:** siRNA, small interefering RNA; miRNA, microRNA; IVT, intravitreal injection; i.v., intravenous; s.c., subcutaneous; FDA, food and drug administration; TRPV1, vanilloid receptor 1; ADRB2, adrenoceptor beta 2; CASP2, caspase 2; RTP801(DDIT4), DNA damage inducible transcript 4; PLK1, polo-like kinase 1; PKN3, protein kinase N3; G12D, glycine to aspartic acid mutation at K-Ras 12^th^ amino acid; VEGFa, vascular endothelial growth factor a; KSP, kinesin spindle protein; TTR, transthyretin; ALAS1, aminolevulinic acid synthase 1; PCSK9, proprotein convertase subtilisin/kexin type 9; HBV, hepatitis B virus; LDH, lactate dehydrogenase.

## Abbreviations

AAT: alpha-1 antitrypsin
ADAR: adenosine deaminase acting on RNA
AE: adverse event
AGO1,2,3,4: argonaute protein 1,2,3,4
ALA: aminolevulinic acid
ALT: alanine transaminase
ALAS1: aminolevulinic acid synthase 1
ANGPT1: angiopoietin
APOB: apolipoprotein B
APA: alternative polyadenylation
APOBEC1: apolipoprotein B mRNA editing enzyme catalytic polypeptide-like 1
ASO: antisense oligonucleotides
AST: aspartate aminotransferase 1
ASPGR1: asialoglycoprotein receptor 1
AURKB: aurora kinase B
A-to-I: adenosine to inosine
BAK1: BCL2 antagonist/killer 1
BCL2: BCL2 apoptosis regulator
BS: binding site
CASP2: caspase 2
CCL2: C-C motif chemokine ligand 2
CCNA2: cyclin A2
CCND1,2: cyclin D1,2
CDC25: cell division cycle 25C
CDH2,6: cadherin 2,6
CDK4,6: cyclin-dependent kinase 4,6
CDI: cyclin-dependent kinase inhibitor
CDK1: cyclin dependent kinase 1
CDS: coding sequence
ceRNA: competing endogenous RNA
cFB: cardiac fibroblast
circRNA: circular RNA
ciRS-7/CDR1-AS: CDR1 antisense RNA
c-KIT: KIT proto-oncogene, receptor tyrosine kinase
CLASH: crosslinking, ligation, and sequencing of hybrids
CLIP: crosslinking and immunoprecipitation
CM: cardiomyocyte
C-to-U: cytosine to uracil
c-MET: MET proto-oncogene, receptor tyrosine kinase
COL1A1: collagen type I alpha 1 chain
COL3A1: collagen type III alpha 1 chain
CPSF5, or CFIm25/NUDT21: cleavage and polyadenylation specificity factor subunit 5
CXCL5: C-X-C motif chemokine ligand 5
DAPK3: death associated protein kinase 3
dCLIP: differential HITS-CLIP
DDIT4 (RTP801): DNA damage inducible transcript 4
DGCR8: DGCR8 microprocessor complex subunit
DICER: dicer 1, ribonuclease III
DROSHA: drosha ribonuclease III
dsRNA: double-stranded RNA
EC: endothelial cell
eCLIP: enhanced CLIP
ECM: extracellular matrix
E2F2,6: E2F transcription factor 2,6
EGFR: epidermal growth factor receptor
EMT: epithelial-mesenchymal-transition
Endo-siRNA: endogenous siRNAs
5EU: 5-ethynyluridine
FEN1: flap structure-specific endonuclease 1
GalNAc: N-acetylgalactosamine
GNA: glycol nucleic acid
HIP1: huntingtin interacting protein 1
HITS-CLIP: High-throughput sequencing of RNAs isolated by CLIP
HMGA2: high mobility group AT-hook 2
HRAS: Hras proto-oncogene, GTPase
HSP70: heat shock protein family A (Hsp70) member 2
HuR: AU-rich element binding protein
ID/g: percent injected dose per gram of tissue
iCLIP: individual nucleotide resolution CLIP
IGF1R: insulin like growth factor 1 receptor
IGFBP: insulin-like growth factor binding protein
linc-MD1: long intergenic non-protein coding RNA, muscle differentiation 1
IL11: interleukin 11
lncRNA: long non-coding RNA
IP: immunoprecipitation
KSP: kinesin spindle protein
LC3 (MAP1LC3A): microtubule associated protein 1 light chain 3 alpha
LDL: low density lipoprotein
LGR4: leucine rich repeat containing G protein-coupled receptor 4
LNA: locked nucleic acid
M^6^A: N6-methyladenosine
MAPK14: mitogen-activated protein kinase 14
m^7^G: 5' methylated guanosine cap
MI: myocardial infarction
MOB1B: MOB kinase activator 1B
mTORC1: mammalian target of rapamycin complex 1
MYC (MYC proto-oncogene): bHLH transcription factor
NANOG: nanog homeobox
nt: nucleotides
NTA: Non-templated nt addition
ORF: Opening reading frame
OCT4 (POU5F1): POU class 5 homeobox 1
p21cip1 (CDKN1A): cyclin dependent kinase inhibitor 1A
p27kip1 (CDKN1B): cyclin dependent kinase inhibitor 1B
p57kip2 (CDKN1C): cyclin dependent kinase inhibitor 1C
PAR-CLIP: photoactivatable ribonucleoside-enhanced CLIP
PAX3: paired box 3
PBG: porphobilinogen
PCSK9: proprotein convertase subtilisin/kexin type 9
PD: pharmacodynamics
PDL1: programmed death 1-ligand 1
PLK1: polo-like kinase 1
PKN3: protein kinase N3
PKR (EIF2AK2): protein kinase R
PUM1,2: pumilio RNA-binding family member 1,2
RACE: rapid amplification of cDNA ends
RBP: RNA binding protein
RISC: RNA-induced silencing complex
RNAi: RNA interference
siRNA: small interefering RNA
miRNA: microRNA
SOX2,9: SRY-box transcription factor 2,9
ss-siRNA: single stranded siRNA
TARBP: TAR RNA-binding protein TARBP
TARBP2: TARBP2 subunit of RISC loading complex
TGF-β1,2: transforming growth factor beta 1,2
TNFa: tumor necrosis factor α
TRPV1 (vanilloid receptor 1): transient receptor potential cation channel subfamily V member 1
TTR: transthyretin
UNA: unlocked nucleic acid
3'-UTR: 3'untranslated region
VCAM1: vascular cell adhesion molecule 1
VEGFa: vascular endothelial growth factor a
VSMC: vascular smooth muscle cells
WBC: white blood cell
YAP1: Yes1 associated transcriptional regulator

## References

[1] Fire A, Xu S, Montgomery MK, Kostas SA, Driver SE, Mello CC (1998). Potent and specific genetic interference by double-stranded RNA in Caenorhabditis elegans. Nature.

[2] Lee RC, Feinbaum RL, Ambros V (1993). The C. elegans heterochronic gene lin-4 encodes small RNAs with antisense complementarity to lin-14. Cell.

[3] Pasquinelli AE, Reinhart BJ, Slack F, Martindale MQ, Kuroda MI, Maller B (2000). Conservation of the sequence and temporal expression of let-7 heterochronic regulatory RNA. Nature.

[4] Liang XH, Sun H, Nichols JG, Crooke ST (2017). RNase H1-Dependent Antisense Oligonucleotides Are Robustly Active in Directing RNA Cleavage in Both the Cytoplasm and the Nucleus. Mol Ther.

[5] Crooke ST, Baker BF, Crooke RM, Liang XH (2021). Antisense technology: an overview and prospectus. Nat Rev Drug Discov.

[6] Krutzfeldt J, Rajewsky N, Braich R, Rajeev KG, Tuschl T, Manoharan M (2005). Silencing of microRNAs *in vivo* with 'antagomirs'. Nature.

[7] Lennox KA, Behlke MA (2011). Chemical modification and design of anti-miRNA oligonucleotides. Gene Ther.

[8] Beierlein JM, McNamee LM, Ledley FD (2017). As Technologies for Nucleotide Therapeutics Mature, Products Emerge. Mol Ther Nucleic Acids.

[9] Setten RL, Rossi JJ, Han SP (2019). The current state and future directions of RNAi-based therapeutics. Nat Rev Drug Discov.

[10] Rupaimoole R, Slack FJ (2017). MicroRNA therapeutics: towards a new era for the management of cancer and other diseases. Nat Rev Drug Discov.

[11] Vidigal JA, Ventura A (2015). The biological functions of miRNAs: lessons from *in vivo* studies. Trends Cell Biol.

[12] Bernstein E, Kim SY, Carmell MA, Murchison EP, Alcorn H, Li MZ (2003). Dicer is essential for mouse development. Nat Genet.

[13] Friedman RC, Farh KK, Burge CB, Bartel DP (2009). Most mammalian mRNAs are conserved targets of microRNAs. Genome Res.

[14] Miranda KC, Huynh T, Tay Y, Ang YS, Tam WL, Thomson AM (2006). A pattern-based method for the identification of MicroRNA binding sites and their corresponding heteroduplexes. Cell.

[15] Kozomara A, Birgaoanu M, Griffiths-Jones S (2019). miRBase: from microRNA sequences to function. Nucleic Acids Res.

[16] Su Y, Wu H, Pavlosky A, Zou LL, Deng X, Zhang ZX (2016). Regulatory non-coding RNA: new instruments in the orchestration of cell death. Cell Death Dis.

[17] Alles J, Fehlmann T, Fischer U, Backes C, Galata V, Minet M (2019). An estimate of the total number of true human miRNAs. Nucleic Acids Res.

[18] Kim VN, Han J, Siomi MC (2009). Biogenesis of small RNAs in animals. Nat Rev Mol Cell Biol.

[19] Ha M, Kim VN (2014). Regulation of microRNA biogenesis. Nat Rev Mol Cell Biol.

[20] Treiber T, Treiber N, Meister G (2019). Regulation of microRNA biogenesis and its crosstalk with other cellular pathways. Nat Rev Mol Cell Biol.

[21] Song R, Hennig GW, Wu Q, Jose C, Zheng H, Yan W (2011). Male germ cells express abundant endogenous siRNAs. Proc Natl Acad Sci U S A.

[22] Watanabe T, Totoki Y, Toyoda A, Kaneda M, Kuramochi-Miyagawa S, Obata Y (2008). Endogenous siRNAs from naturally formed dsRNAs regulate transcripts in mouse oocytes. Nature.

[23] Llave C, Kasschau KD, Rector MA, Carrington JC (2002). Endogenous and silencing-associated small RNAs in plants. Plant Cell.

[24] Carlile M, Nalbant P, Preston-Fayers K, McHaffie GS, Werner A (2008). Processing of naturally occurring sense/antisense transcripts of the vertebrate Slc34a gene into short RNAs. Physiol Genomics.

[25] Carthew RW, Sontheimer EJ (2009). Origins and Mechanisms of miRNAs and siRNAs. Cell.

[26] Li ML, Weng KF, Shih SR, Brewer G (2016). The evolving world of small RNAs from RNA viruses. Wiley Interdiscip Rev RNA.

[27] Lee YS, Nakahara K, Pham JW, Kim K, He Z, Sontheimer EJ (2004). Distinct roles for Drosophila Dicer-1 and Dicer-2 in the siRNA/miRNA silencing pathways. Cell.

[28] Tabara H, Sarkissian M, Kelly WG, Fleenor J, Grishok A, Timmons L (1999). The rde-1 gene, RNA interference, and transposon silencing in C. elegans. Cell.

[29] Secombes CJ, Zou J (2017). Evolution of Interferons and Interferon Receptors. Front Immunol.

[30] Gantier MP, Williams BR (2007). The response of mammalian cells to double-stranded RNA. Cytokine Growth Factor Rev.

[31] Kawamata T, Tomari Y (2010). Making RISC. Trends Biochem Sci.

[32] Elkayam E, Kuhn CD, Tocilj A, Haase AD, Greene EM, Hannon GJ (2012). The structure of human argonaute-2 in complex with miR-20a. Cell.

[33] Schirle NT, Kinberger GA, Murray HF, Lima WF, Prakash TP, MacRae IJ (2016). Structural Analysis of Human Argonaute-2 Bound to a Modified siRNA Guide. J Am Chem Soc.

[34] Wang HW, Noland C, Siridechadilok B, Taylor DW, Ma E, Felderer K (2009). Structural insights into RNA processing by the human RISC-loading complex. Nat Struct Mol Biol.

[35] Schwarz DS, Hutvagner G, Du T, Xu Z, Aronin N, Zamore PD (2003). Asymmetry in the assembly of the RNAi enzyme complex. Cell.

[36] Khvorova A, Reynolds A, Jayasena SD (2003). Functional siRNAs and miRNAs exhibit strand bias. Cell.

[37] Sanei M, Chen X (2015). Mechanisms of microRNA turnover. Curr Opin Plant Biol.

[38] Nielsen CB, Shomron N, Sandberg R, Hornstein E, Kitzman J, Burge CB (2007). Determinants of targeting by endogenous and exogenous microRNAs and siRNAs. RNA.

[39] Moore MJ, Scheel TK, Luna JM, Park CY, Fak JJ, Nishiuchi E (2015). miRNA-target chimeras reveal miRNA 3'-end pairing as a major determinant of Argonaute target specificity. Nat Commun.

[40] Broughton JP, Lovci MT, Huang JL, Yeo GW, Pasquinelli AE (2016). Pairing beyond the Seed Supports MicroRNA Targeting Specificity. Mol Cell.

[41] Hutvagner G, Simard MJ (2008). Argonaute proteins: key players in RNA silencing. Nat Rev Mol Cell Biol.

[42] Niaz S (2018). The AGO proteins: an overview. Biol Chem.

[43] Jonas S, Izaurralde E (2015). Towards a molecular understanding of microRNA-mediated gene silencing. Nat Rev Genet.

[44] Reichholf B, Herzog VA, Fasching N, Manzenreither RA, Sowemimo I, Ameres SL (2019). Time-Resolved Small RNA Sequencing Unravels the Molecular Principles of MicroRNA Homeostasis. Mol Cell.

[45] Kingston ER, Bartel DP (2019). Global analyses of the dynamics of mammalian microRNA metabolism. Genome Res.

[46] Flores O, Kennedy EM, Skalsky RL, Cullen BR (2014). Differential RISC association of endogenous human microRNAs predicts their inhibitory potential. Nucleic Acids Res.

[47] Mayya VK, Duchaine TF (2015). On the availability of microRNA-induced silencing complexes, saturation of microRNA-binding sites and stoichiometry. Nucleic Acids Res.

[48] Wittrup A, Lieberman J (2015). Knocking down disease: a progress report on siRNA therapeutics. Nat Rev Genet.

[49] Elbashir SM, Lendeckel W, Tuschl T (2001). RNA interference is mediated by 21- and 22-nucleotide RNAs. Genes Dev.

[50] Rivas FV, Tolia NH, Song JJ, Aragon JP, Liu J, Hannon GJ (2005). Purified Argonaute2 and an siRNA form recombinant human RISC. Nat Struct Mol Biol.

[51] Elbashir SM, Harborth J, Lendeckel W, Yalcin A, Weber K, Tuschl T (2001). Duplexes of 21-nucleotide RNAs mediate RNA interference in cultured mammalian cells. Nature.

[52] Reynolds A, Anderson EM, Vermeulen A, Fedorov Y, Robinson K, Leake D (2006). Induction of the interferon response by siRNA is cell type- and duplex length-dependent. RNA.

[53] Kim DH, Behlke MA, Rose SD, Chang MS, Choi S, Rossi JJ (2005). Synthetic dsRNA Dicer substrates enhance RNAi potency and efficacy. Nat Biotechnol.

[54] Snead NM, Wu X, Li A, Cui Q, Sakurai K, Burnett JC (2013). Molecular basis for improved gene silencing by Dicer substrate interfering RNA compared with other siRNA variants. Nucleic Acids Res.

[55] Sakurai K, Amarzguioui M, Kim DH, Alluin J, Heale B, Song MS (2011). A role for human Dicer in pre-RISC loading of siRNAs. Nucleic Acids Res.

[56] Collingwood MA, Rose SD, Huang L, Hillier C, Amarzguioui M, Wiiger MT (2008). Chemical modification patterns compatible with high potency dicer-substrate small interfering RNAs. Oligonucleotides.

[57] Martinez J, Patkaniowska A, Urlaub H, Luhrmann R, Tuschl T (2002). Single-stranded antisense siRNAs guide target RNA cleavage in RNAi. Cell.

[58] Lima WF, Prakash TP, Murray HM, Kinberger GA, Li W, Chappell AE (2012). Single-stranded siRNAs activate RNAi in animals. Cell.

[59] Xu Y, Linde A, Larsson O, Thormeyer D, Elmen J, Wahlestedt C (2004). Functional comparison of single- and double-stranded siRNAs in mammalian cells. Biochem Biophys Res Commun.

[60] Pendergraff HM, Debacker AJ, Watts JK (2016). Single-Stranded Silencing RNAs: Hit Rate and Chemical Modification. Nucleic Acid Ther.

[61] Yu D, Pendergraff H, Liu J, Kordasiewicz HB, Cleveland DW, Swayze EE (2012). Single-stranded RNAs use RNAi to potently and allele-selectively inhibit mutant huntingtin expression. Cell.

[62] Brummelkamp TR, Bernards R, Agami R (2002). A system for stable expression of short interfering RNAs in mammalian cells. Science.

[63] Esrick EB, Lehmann LE, Biffi A, Achebe M, Brendel C, Ciuculescu MF (2021). Post-Transcriptional Genetic Silencing of BCL11A to Treat Sickle Cell Disease. N Engl J Med.

[64] Grimm D, Streetz KL, Jopling CL, Storm TA, Pandey K, Davis CR (2006). Fatality in mice due to oversaturation of cellular microRNA/short hairpin RNA pathways. Nature.

[65] Jin X, Sun T, Zhao C, Zheng Y, Zhang Y, Cai W (2012). Strand antagonism in RNAi: an explanation of differences in potency between intracellularly expressed siRNA and shRNA. Nucleic Acids Res.

[66] Chen PY, Weinmann L, Gaidatzis D, Pei Y, Zavolan M, Tuschl T (2008). Strand-specific 5'-O-methylation of siRNA duplexes controls guide strand selection and targeting specificity. RNA.

[67] Mobergslien A, Sioud M (2015). A facile method for interfering with off-target silencing mediated by the sense strand. Methods Mol Biol.

[68] Zheng J, Zhang L, Zhang J, Wang X, Ye K, Xi Z (2013). Single modification at position 14 of siRNA strand abolishes its gene-silencing activity by decreasing both RISC loading and target degradation. FASEB J.

[69] Kumar P, Parmar RG, Brown CR, Willoughby JLS, Foster DJ, Babu IR (2019). 5'-Morpholino modification of the sense strand of an siRNA makes it a more effective passenger. Chem Commun (Camb).

[70] Bramsen JB, Kjems J (2012). Development of Therapeutic-Grade Small Interfering RNAs by Chemical Engineering. Front Genet.

[71] Fakhr E, Zare F, Teimoori-Toolabi L (2016). Precise and efficient siRNA design: a key point in competent gene silencing. Cancer Gene Ther.

[72] Pink RC, Samuel P, Massa D, Caley DP, Brooks SA, Carter DR (2015). The passenger strand, miR-21-3p, plays a role in mediating cisplatin resistance in ovarian cancer cells. Gynecol Oncol.

[73] Schober A, Nazari-Jahantigh M, Wei Y, Bidzhekov K, Gremse F, Grommes J (2014). MicroRNA-126-5p promotes endothelial proliferation and limits atherosclerosis by suppressing Dlk1. Nat Med.

[74] Zhou Q, Anderson C, Hanus J, Zhao F, Ma J, Yoshimura A (2016). Strand and Cell Type-specific Function of microRNA-126 in Angiogenesis. Mol Ther.

[75] Ferino A, Miglietta G, Picco R, Vogel S, Wengel J, Xodo LE (2018). MicroRNA therapeutics: design of single-stranded miR-216b mimics to target KRAS in pancreatic cancer cells. RNA Biol.

[76] Chorn G, Klein-McDowell M, Zhao L, Saunders MA, Flanagan WM, Willingham AT (2012). Single-stranded microRNA mimics. RNA.

[77] Matsui M, Prakash TP, Corey DR (2016). Argonaute 2-dependent Regulation of Gene Expression by Single-stranded miRNA Mimics. Mol Ther.

[78] van Rooij E, Kauppinen S (2014). Development of microRNA therapeutics is coming of age. EMBO Mol Med.

[79] Sokilde R, Newie I, Persson H, Borg A, Rovira C (2015). Passenger strand loading in overexpression experiments using microRNA mimics. RNA Biol.

[80] Reid G, Pel ME, Kirschner MB, Cheng YY, Mugridge N, Weiss J (2013). Restoring expression of miR-16: a novel approach to therapy for malignant pleural mesothelioma. Ann Oncol.

[81] Montgomery RL, Yu G, Latimer PA, Stack C, Robinson K, Dalby CM (2014). MicroRNA mimicry blocks pulmonary fibrosis. EMBO Mol Med.

[82] Git A (2012). Research tools: A recipe for disaster. Nature.

[83] Jackson AL, Bartz SR, Schelter J, Kobayashi SV, Burchard J, Mao M (2003). Expression profiling reveals off-target gene regulation by RNAi. Nat Biotechnol.

[84] Doench JG, Petersen CP, Sharp PA (2003). siRNAs can function as miRNAs. Genes Dev.

[85] Jackson AL, Burchard J, Schelter J, Chau BN, Cleary M, Lim L (2006). Widespread siRNA "off-target" transcript silencing mediated by seed region sequence complementarity. RNA.

[86] Janas MM, Schlegel MK, Harbison CE, Yilmaz VO, Jiang Y, Parmar R (2018). Selection of GalNAc-conjugated siRNAs with limited off-target-driven rat hepatotoxicity. Nat Commun.

[87] Naito Y, Yoshimura J, Morishita S, Ui-Tei K (2009). siDirect 2.0: updated software for designing functional siRNA with reduced seed-dependent off-target effect. BMC Bioinformatics.

[88] Lin X, Ruan X, Anderson MG, McDowell JA, Kroeger PE, Fesik SW (2005). siRNA-mediated off-target gene silencing triggered by a 7 nt complementation. Nucleic Acids Res.

[89] Fedorov Y, Anderson EM, Birmingham A, Reynolds A, Karpilow J, Robinson K (2006). Off-target effects by siRNA can induce toxic phenotype. RNA.

[90] Daga N, Eicher S, Kannan A, Casanova A, Low SH, Kreibich S (2018). Growth-restricting effects of siRNA transfections: a largely deterministic combination of off-target binding and hybridization-independent competition. Nucleic Acids Res.

[91] Tadin-Strapps M, Peterson LB, Cumiskey AM, Rosa RL, Mendoza VH, Castro-Perez J (2011). siRNA-induced liver ApoB knockdown lowers serum LDL-cholesterol in a mouse model with human-like serum lipids. J Lipid Res.

[92] Frank-Kamenetsky M, Grefhorst A, Anderson NN, Racie TS, Bramlage B, Akinc A (2008). Therapeutic RNAi targeting PCSK9 acutely lowers plasma cholesterol in rodents and LDL cholesterol in nonhuman primates. Proc Natl Acad Sci U S A.

[93] Hannus M, Beitzinger M, Engelmann JC, Weickert MT, Spang R, Hannus S (2014). siPools: highly complex but accurately defined siRNA pools eliminate off-target effects. Nucleic Acids Res.

[94] Jackson AL, Burchard J, Leake D, Reynolds A, Schelter J, Guo J (2006). Position-specific chemical modification of siRNAs reduces "off-target" transcript silencing. RNA.

[95] Bramsen JB, Pakula MM, Hansen TB, Bus C, Langkjaer N, Odadzic D (2010). A screen of chemical modifications identifies position-specific modification by UNA to most potently reduce siRNA off-target effects. Nucleic Acids Res.

[96] Dua P, Yoo JW, Kim S, Lee DK (2011). Modified siRNA structure with a single nucleotide bulge overcomes conventional siRNA-mediated off-target silencing. Mol Ther.

[97] Lee HS, Seok H, Lee DH, Ham J, Lee W, Youm EM (2015). Abasic pivot substitution harnesses target specificity of RNA interference. Nat Commun.

[98] Ui-Tei K, Naito Y, Nishi K, Juni A, Saigo K (2008). Thermodynamic stability and Watson-Crick base pairing in the seed duplex are major determinants of the efficiency of the siRNA-based off-target effect. Nucleic Acids Res.

[99] Gu S, Zhang Y, Jin L, Huang Y, Zhang F, Bassik MC (2014). Weak base pairing in both seed and 3' regions reduces RNAi off-targets and enhances si/shRNA designs. Nucleic Acids Res.

[100] Seok H, Lee H, Jang ES, Chi SW (2018). Evaluation and control of miRNA-like off-target repression for RNA interference. Cell Mol Life Sci.

[101] Burchard J, Jackson AL, Malkov V, Needham RH, Tan Y, Bartz SR (2009). MicroRNA-like off-target transcript regulation by siRNAs is species specific. RNA.

[102] Soutschek J, Akinc A, Bramlage B, Charisse K, Constien R, Donoghue M (2004). Therapeutic silencing of an endogenous gene by systemic administration of modified siRNAs. Nature.

[103] Mukai H, Hatanaka K, Yagi N, Warashina S, Zouda M, Takahashi M (2019). Pharmacokinetic evaluation of liposomal nanoparticle-encapsulated nucleic acid drug: A combined study of dynamic PET imaging and LC/MS/MS analysis. J Control Release.

[104] Arnold AS, Tang YL, Qian K, Shen L, Valencia V, Phillips MI (2007). Specific beta1-adrenergic receptor silencing with small interfering RNA lowers high blood pressure and improves cardiac function in myocardial ischemia. J Hypertens.

[105] Mikhaylova M, Stasinopoulos I, Kato Y, Artemov D, Bhujwalla ZM (2009). Imaging of cationic multifunctional liposome-mediated delivery of COX-2 siRNA. Cancer Gene Ther.

[106] Lewis DL, Hagstrom JE, Loomis AG, Wolff JA, Herweijer H (2002). Efficient delivery of siRNA for inhibition of gene expression in postnatal mice. Nat Genet.

[107] Nascimento AV, Gattacceca F, Singh A, Bousbaa H, Ferreira D, Sarmento B (2016). Biodistribution and pharmacokinetics of Mad2 siRNA-loaded EGFR-targeted chitosan nanoparticles in cisplatin sensitive and resistant lung cancer models. Nanomedicine (Lond).

[108] Kowalski PS, Zwiers PJ, Morselt HW, Kuldo JM, Leus NG, Ruiters MH (2014). Anti-VCAM-1 SAINT-O-Somes enable endothelial-specific delivery of siRNA and downregulation of inflammatory genes in activated endothelium *in vivo*. J Control Release.

[109] Leus NG, Morselt HW, Zwiers PJ, Kowalski PS, Ruiters MH, Molema G (2014). VCAM-1 specific PEGylated SAINT-based lipoplexes deliver siRNA to activated endothelium *in vivo* but do not attenuate target gene expression. Int J Pharm.

[110] Huang YH, Peng W, Furuuchi N, Gerhart J, Rhodes K, Mukherjee N (2016). Delivery of Therapeutics Targeting the mRNA-Binding Protein HuR Using 3DNA Nanocarriers Suppresses Ovarian Tumor Growth. Cancer Res.

[111] Lu W, Zhang G, Zhang R, Flores LG 2nd, Huang Q, Gelovani JG (2010). Tumor site-specific silencing of NF-kappaB p65 by targeted hollow gold nanosphere-mediated photothermal transfection. Cancer Res.

[112] Matkovich SJ, Wang W, Tu Y, Eschenbacher WH, Dorn LE, Condorelli G (2010). MicroRNA-133a protects against myocardial fibrosis and modulates electrical repolarization without affecting hypertrophy in pressure-overloaded adult hearts. Circ Res.

[113] Zhou Y, Richards AM, Wang P (2019). MicroRNA-221 Is Cardioprotective and Anti-fibrotic in a Rat Model of Myocardial Infarction. Mol Ther Nucleic Acids.

[114] Tian Y, Liu Y, Wang T, Zhou N, Kong J, Chen L (2015). A microRNA-Hippo pathway that promotes cardiomyocyte proliferation and cardiac regeneration in mice. Sci Transl Med.

[115] Raza U, Saatci O, Uhlmann S, Ansari SA, Eyupoglu E, Yurdusev E (2016). The miR-644a/CTBP1/p53 axis suppresses drug resistance by simultaneous inhibition of cell survival and epithelial-mesenchymal transition in breast cancer. Oncotarget.

[116] Erhard F, Haas J, Lieber D, Malterer G, Jaskiewicz L, Zavolan M (2014). Widespread context dependency of microRNA-mediated regulation. Genome Res.

[117] Sood P, Krek A, Zavolan M, Macino G, Rajewsky N (2006). Cell-type-specific signatures of microRNAs on target mRNA expression. Proc Natl Acad Sci U S A.

[118] Ludwig N, Leidinger P, Becker K, Backes C, Fehlmann T, Pallasch C (2016). Distribution of miRNA expression across human tissues. Nucleic Acids Res.

[119] Rogg EM, Abplanalp WT, Bischof C, John D, Schulz MH, Krishnan J (2018). Analysis of Cell Type-Specific Effects of MicroRNA-92a Provides Novel Insights Into Target Regulation and Mechanism of Action. Circulation.

[120] Liu X, Cheng Y, Yang J, Xu L, Zhang C (2012). Cell-specific effects of miR-221/222 in vessels: molecular mechanism and therapeutic application. J Mol Cell Cardiol.

[121] Zhou Y, Shiok TC, Richards AM, Wang P (2018). MicroRNA-101a suppresses fibrotic programming in isolated cardiac fibroblasts and *in vivo* fibrosis following trans-aortic constriction. J Mol Cell Cardiol.

[122] Nam JW, Rissland OS, Koppstein D, Abreu-Goodger C, Jan CH, Agarwal V (2014). Global analyses of the effect of different cellular contexts on microRNA targeting. Mol Cell.

[123] Lim LP, Lau NC, Garrett-Engele P, Grimson A, Schelter JM, Castle J (2005). Microarray analysis shows that some microRNAs downregulate large numbers of target mRNAs. Nature.

[124] Baek D, Villen J, Shin C, Camargo FD, Gygi SP, Bartel DP (2008). The impact of microRNAs on protein output. Nature.

[125] Selbach M, Schwanhausser B, Thierfelder N, Fang Z, Khanin R, Rajewsky N (2008). Widespread changes in protein synthesis induced by microRNAs. Nature.

[126] Johnson SM, Grosshans H, Shingara J, Byrom M, Jarvis R, Cheng A (2005). RAS is regulated by the let-7 microRNA family. Cell.

[127] Lee YS, Dutta A (2007). The tumor suppressor microRNA let-7 represses the HMGA2 oncogene. Genes Dev.

[128] Yu F, Yao H, Zhu P, Zhang X, Pan Q, Gong C (2007). let-7 regulates self renewal and tumorigenicity of breast cancer cells. Cell.

[129] Barh D, Malhotra R, Ravi B, Sindhurani P (2010). MicroRNA let-7: an emerging next-generation cancer therapeutic. Curr Oncol.

[130] Icli B, Wu W, Ozdemir D, Li H, Haemmig S, Liu X (2019). MicroRNA-135a-3p regulates angiogenesis and tissue repair by targeting p38 signaling in endothelial cells. FASEB J.

[131] Altuvia Y, Landgraf P, Lithwick G, Elefant N, Pfeffer S, Aravin A (2005). Clustering and conservation patterns of human microRNAs. Nucleic Acids Res.

[132] Peter ME (2010). Targeting of mRNAs by multiple miRNAs: the next step. Oncogene.

[133] Kim YK, Yu J, Han TS, Park SY, Namkoong B, Kim DH (2009). Functional links between clustered microRNAs: suppression of cell-cycle inhibitors by microRNA clusters in gastric cancer. Nucleic Acids Res.

[134] Zhang J, Pham VVH, Liu L, Xu T, Truong B, Li J (2019). Identifying miRNA synergism using multiple-intervention causal inference. BMC Bioinformatics.

[135] Bandi N, Vassella E (2011). miR-34a and miR-15a/16 are co-regulated in non-small cell lung cancer and control cell cycle progression in a synergistic and Rb-dependent manner. Mol Cancer.

[136] Landgraf P, Rusu M, Sheridan R, Sewer A, Iovino N, Aravin A (2007). A mammalian microRNA expression atlas based on small RNA library sequencing. Cell.

[137] Olejniczak M, Kotowska-Zimmer A, Krzyzosiak W (2018). Stress-induced changes in miRNA biogenesis and functioning. Cell Mol Life Sci.

[138] Telonis AG, Magee R, Loher P, Chervoneva I, Londin E, Rigoutsos I (2017). Knowledge about the presence or absence of miRNA isoforms (isomiRs) can successfully discriminate amongst 32 TCGA cancer types. Nucleic Acids Res.

[139] Wu CW, Evans JM, Huang S, Mahoney DW, Dukek BA, Taylor WR (2018). A Comprehensive Approach to Sequence-oriented IsomiR annotation (CASMIR): demonstration with IsomiR profiling in colorectal neoplasia. BMC Genomics.

[140] Marti E, Pantano L, Banez-Coronel M, Llorens F, Minones-Moyano E, Porta S (2010). A myriad of miRNA variants in control and Huntington's disease brain regions detected by massively parallel sequencing. Nucleic Acids Res.

[141] Pantano L, Friedlander MR, Escaramis G, Lizano E, Pallares-Albanell J, Ferrer I (2016). Specific small-RNA signatures in the amygdala at premotor and motor stages of Parkinson's disease revealed by deep sequencing analysis. Bioinformatics.

[142] Koppers-Lalic D, Hackenberg M, de Menezes R, Misovic B, Wachalska M, Geldof A (2016). Noninvasive prostate cancer detection by measuring miRNA variants (isomiRs) in urine extracellular vesicles. Oncotarget.

[143] Wang S, Xu Y, Li M, Tu J, Lu Z (2016). Dysregulation of miRNA isoform level at 5' end in Alzheimer's disease. Gene.

[144] van der Kwast R, Woudenberg T, Quax PHA, Nossent AY (2020). MicroRNA-411 and Its 5'-IsomiR Have Distinct Targets and Functions and Are Differentially Regulated in the Vasculature under Ischemia. Mol Ther.

[145] Mi S, Cai T, Hu Y, Chen Y, Hodges E, Ni F (2008). Sorting of small RNAs into Arabidopsis argonaute complexes is directed by the 5' terminal nucleotide. Cell.

[146] Neilsen CT, Goodall GJ, Bracken CP (2012). IsomiRs-the overlooked repertoire in the dynamic microRNAome. Trends Genet.

[147] Yu F, Pillman KA, Neilsen CT, Toubia J, Lawrence DM, Tsykin A (2017). Naturally existing isoforms of miR-222 have distinct functions. Nucleic Acids Res.

[148] Wu H, Ye C, Ramirez D, Manjunath N (2009). Alternative processing of primary microRNA transcripts by Drosha generates 5' end variation of mature microRNA. PLoS One.

[149] Fukunaga R, Han BW, Hung JH, Xu J, Weng Z, Zamore PD (2012). Dicer Partner Proteins Tune the Length of Mature miRNAs in Flies and Mammals. Cell.

[150] Luciano DJ, Mirsky H, Vendetti NJ, Maas S (2004). RNA editing of a miRNA precursor. RNA.

[151] Bass BL, Nishikura K, Keller W, Seeburg PH, Emeson RB, O'Connell MA (1997). A standardized nomenclature for adenosine deaminases that act on RNA. RNA.

[152] Rosenberg BR, Hamilton CE, Mwangi MM, Dewell S, Papavasiliou FN (2011). Transcriptome-wide sequencing reveals numerous APOBEC1 mRNA-editing targets in transcript 3' UTRs. Nat Struct Mol Biol.

[153] Peng Z, Cheng Y, Tan BC, Kang L, Tian Z, Zhu Y (2012). Comprehensive analysis of RNA-Seq data reveals extensive RNA editing in a human transcriptome. Nat Biotechnol.

[154] Ekdahl Y, Farahani HS, Behm M, Lagergren J, Ohman M (2012). A-to-I editing of microRNAs in the mammalian brain increases during development. Genome Res.

[155] Kawahara Y, Megraw M, Kreider E, Iizasa H, Valente L, Hatzigeorgiou AG (2008). Frequency and fate of microRNA editing in human brain. Nucleic Acids Res.

[156] Nishikura K (2016). A-to-I editing of coding and non-coding RNAs by ADARs. Nat Rev Mol Cell Biol.

[157] Boele J, Persson H, Shin JW, Ishizu Y, Newie IS, Sokilde R (2014). PAPD5-mediated 3' adenylation and subsequent degradation of miR-21 is disrupted in proliferative disease. Proc Natl Acad Sci U S A.

[158] Gutierrez-Vazquez C, Enright AJ, Rodriguez-Galan A, Perez-Garcia A, Collier P, Jones MR (2017). 3' Uridylation controls mature microRNA turnover during CD4 T-cell activation. RNA.

[159] Jones MR, Quinton LJ, Blahna MT, Neilson JR, Fu S, Ivanov AR (2009). Zcchc11-dependent uridylation of microRNA directs cytokine expression. Nat Cell Biol.

[160] Jin HY, Gonzalez-Martin A, Miletic AV, Lai M, Knight S, Sabouri-Ghomi M (2015). Transfection of microRNA Mimics Should Be Used with Caution. Front Genet.

[161] Schamberger A, Orban TI (2014). 3' IsomiR species and DNA contamination influence reliable quantification of microRNAs by stem-loop quantitative PCR. PLoS One.

[162] Magee R, Telonis AG, Cherlin T, Rigoutsos I, Londin E (2017). Assessment of isomiR Discrimination Using Commercial qPCR Methods. Noncoding RNA.

[163] Honda S, Kirino Y (2015). Dumbbell-PCR: a method to quantify specific small RNA variants with a single nucleotide resolution at terminal sequences. Nucleic Acids Res.

[164] Androvic P, Valihrach L, Elling J, Sjoback R, Kubista M (2017). Two-tailed RT-qPCR: a novel method for highly accurate miRNA quantification. Nucleic Acids Res.

[165] Gerstberger S, Hafner M, Tuschl T (2014). A census of human RNA-binding proteins. Nat Rev Genet.

[166] He L, He X, Lim LP, de Stanchina E, Xuan Z, Liang Y (2007). A microRNA component of the p53 tumour suppressor network. Nature.

[167] Xiao R, Chen JY, Liang Z, Luo D, Chen G, Lu ZJ (2019). Pervasive Chromatin-RNA Binding Protein Interactions Enable RNA-Based Regulation of Transcription. Cell.

[168] Treiber T, Treiber N, Plessmann U, Harlander S, Daiss JL, Eichner N (2017). A Compendium of RNA-Binding Proteins that Regulate MicroRNA Biogenesis. Mol Cell.

[169] Nussbacher JK, Yeo GW (2018). Systematic Discovery of RNA Binding Proteins that Regulate MicroRNA Levels. Mol Cell.

[170] Mazan-Mamczarz K, Galban S, Lopez de Silanes I, Martindale JL, Atasoy U, Keene JD (2003). RNA-binding protein HuR enhances p53 translation in response to ultraviolet light irradiation. Proc Natl Acad Sci U S A.

[171] Meisner NC, Filipowicz W (2011). Properties of the Regulatory RNA-Binding Protein HuR and its Role in Controlling miRNA Repression. Adv Exp Med Biol.

[172] Srikantan S, Tominaga K, Gorospe M (2012). Functional interplay between RNA-binding protein HuR and microRNAs. Curr Protein Pept Sci.

[173] Fan XC, Steitz JA (1998). Overexpression of HuR, a nuclear-cytoplasmic shuttling protein, increases the *in vivo* stability of ARE-containing mRNAs. EMBO J.

[174] Kim HH, Kuwano Y, Srikantan S, Lee EK, Martindale JL, Gorospe M (2009). HuR recruits let-7/RISC to repress c-Myc expression. Genes Dev.

[175] Epis MR, Barker A, Giles KM, Beveridge DJ, Leedman PJ (2011). The RNA-binding protein HuR opposes the repression of ERBB-2 gene expression by microRNA miR-331-3p in prostate cancer cells. J Biol Chem.

[176] Galgano A, Forrer M, Jaskiewicz L, Kanitz A, Zavolan M, Gerber AP (2008). Comparative analysis of mRNA targets for human PUF-family proteins suggests extensive interaction with the miRNA regulatory system. PLoS One.

[177] le Sage C, Nagel R, Egan DA, Schrier M, Mesman E, Mangiola A (2007). Regulation of the p27(Kip1) tumor suppressor by miR-221 and miR-222 promotes cancer cell proliferation. EMBO J.

[178] Liu X, Cheng Y, Zhang S, Lin Y, Yang J, Zhang C (2009). A necessary role of miR-221 and miR-222 in vascular smooth muscle cell proliferation and neointimal hyperplasia. Circ Res.

[179] Kedde M, van Kouwenhove M, Zwart W, Oude Vrielink JA, Elkon R, Agami R (2010). A Pumilio-induced RNA structure switch in p27-3' UTR controls miR-221 and miR-222 accessibility. Nat Cell Biol.

[180] Zhou Y, Richards AM, Wang P (2019). Loss of pumilio 1 phosphorylation turns off the regulation of miRNA-221 on gene p27kip1 in the heart. In: Eur Heart J.

[181] Leibovich L, Mandel-Gutfreund Y, Yakhini Z (2010). A structural-based statistical approach suggests a cooperative activity of PUM1 and miR-410 in human 3'-untranslated regions. Silence.

[182] Reinhart BJ, Slack FJ, Basson M, Pasquinelli AE, Bettinger JC, Rougvie AE (2000). The 21-nucleotide let-7 RNA regulates developmental timing in Caenorhabditis elegans. Nature.

[183] Xie X, Lu J, Kulbokas EJ, Golub TR, Mootha V, Lindblad-Toh K (2005). Systematic discovery of regulatory motifs in human promoters and 3' UTRs by comparison of several mammals. Nature.

[184] Lai EC (2002). Micro RNAs are complementary to 3' UTR sequence motifs that mediate negative post-transcriptional regulation. Nat Genet.

[185] Proudfoot NJ (2011). Ending the message: poly(A) signals then and now. Genes Dev.

[186] Beaudoing E, Freier S, Wyatt JR, Claverie JM, Gautheret D (2000). Patterns of variant polyadenylation signal usage in human genes. Genome Res.

[187] Anvar SY, Allard G, Tseng E, Sheynkman GM, de Klerk E, Vermaat M (2018). Full-length mRNA sequencing uncovers a widespread coupling between transcription initiation and mRNA processing. Genome Biol.

[188] Hardy JG, Norbury CJ (2016). Cleavage factor Im (CFIm) as a regulator of alternative polyadenylation. Biochem Soc Trans.

[189] Tian B, Hu J, Zhang H, Lutz CS (2005). A large-scale analysis of mRNA polyadenylation of human and mouse genes. Nucleic Acids Res.

[190] Chen X, Zhang JX, Luo JH, Wu S, Yuan GJ, Ma NF (2018). CSTF2-Induced Shortening of the RAC1 3'UTR Promotes the Pathogenesis of Urothelial Carcinoma of the Bladder. Cancer Res.

[191] Andres SF, Williams KN, Plesset JB, Headd JJ, Mizuno R, Chatterji P (2019). IMP1 3' UTR shortening enhances metastatic burden in colorectal cancer. Carcinogenesis.

[192] Chu Y, Elrod N, Wang C, Li L, Chen T, Routh A (2019). Nudt21 regulates the alternative polyadenylation of Pak1 and is predictive in the prognosis of glioblastoma patients. Oncogene.

[193] Mayr C, Bartel DP (2009). Widespread shortening of 3'UTRs by alternative cleavage and polyadenylation activates oncogenes in cancer cells. Cell.

[194] Weng T, Ko J, Masamha CP, Xia Z, Xiang Y, Chen NY (2019). Cleavage factor 25 deregulation contributes to pulmonary fibrosis through alternative polyadenylation. J Clin Invest.

[195] Zhou Z, Qu J, He L, Zhu Y, Yang SZ, Zhang F (2020). Stiff matrix instigates type I collagen biogenesis by mammalian cleavage factor I complex-mediated alternative polyadenylation. JCI Insight.

[196] Weng T, Huang J, Wagner EJ, Ko J, Wu M, Wareing NE (2020). Downregulation of CFIm25 amplifies dermal fibrosis through alternative polyadenylation. J Exp Med.

[197] Soetanto R, Hynes CJ, Patel HR, Humphreys DT, Evers M, Duan G (2016). Role of miRNAs and alternative mRNA 3'-end cleavage and polyadenylation of their mRNA targets in cardiomyocyte hypertrophy. Biochim Biophys Acta.

[198] Zheng D, Wang R, Ding Q, Wang T, Xie B, Wei L (2018). Cellular stress alters 3'UTR landscape through alternative polyadenylation and isoform-specific degradation. Nat Commun.

[199] Chang JW, Zhang W, Yeh HS, de Jong EP, Jun S, Kim KH (2015). mRNA 3'-UTR shortening is a molecular signature of mTORC1 activation. Nat Commun.

[200] Pai AA, Baharian G, Page Sabourin A, Brinkworth JF, Nedelec Y, F (2016). Widespread Shortening of 3' Untranslated Regions and Increased Exon Inclusion Are Evolutionarily Conserved Features of Innate Immune Responses to Infection. PLoS Genet.

[201] Elkon R, Ugalde AP, Agami R (2013). Alternative cleavage and polyadenylation: extent, regulation and function. Nat Rev Genet.

[202] de Morree A, Klein JDD, Gan Q, Farup J, Urtasun A, Kanugovi A (2019). Alternative polyadenylation of Pax3 controls muscle stem cell fate and muscle function. Science.

[203] Tranter M, Helsley RN, Paulding WR, McGuinness M, Brokamp C, Haar L (2011). Coordinated post-transcriptional regulation of Hsp70.3 gene expression by microRNA and alternative polyadenylation. J Biol Chem.

[204] Masamha CP, Xia Z, Peart N, Collum S, Li W, Wagner EJ (2016). CFIm25 regulates glutaminase alternative terminal exon definition to modulate miR-23 function. RNA.

[205] Eisen TJ, Eichhorn SW, Subtelny AO, Bartel DP (2020). MicroRNAs Cause Accelerated Decay of Short-Tailed Target mRNAs. Mol Cell.

[206] Grimson A, Farh KK, Johnston WK, Garrett-Engele P, Lim LP, Bartel DP (2007). MicroRNA targeting specificity in mammals: determinants beyond seed pairing. Mol Cell.

[207] Shulman ED, Elkon R (2019). Cell-type-specific analysis of alternative polyadenylation using single-cell transcriptomics data. Nucleic Acids Res.

[208] Thomson DW, Dinger ME (2016). Endogenous microRNA sponges: evidence and controversy. Nat Rev Genet.

[209] Ebert MS, Sharp PA (2010). Emerging roles for natural microRNA sponges. Curr Biol.

[210] Maass PG, Glazar P, Memczak S, Dittmar G, Hollfinger I, Schreyer L (2017). A map of human circular RNAs in clinically relevant tissues. J Mol Med.

[211] Hansen TB, Jensen TI, Clausen BH, Bramsen JB, Finsen B, Damgaard CK (2013). Natural RNA circles function as efficient microRNA sponges. Nature.

[212] Memczak S, Jens M, Elefsinioti A, Torti F, Krueger J, Rybak A (2013). Circular RNAs are a large class of animal RNAs with regulatory potency. Nature.

[213] Kapranov P, Cheng J, Dike S, Nix DA, Duttagupta R, Willingham AT (2007). RNA maps reveal new RNA classes and a possible function for pervasive transcription. Science.

[214] Ma L, Cao J, Liu L, Du Q, Li Z, Zou D (2019). LncBook: a curated knowledgebase of human long non-coding RNAs. Nucleic Acids Res.

[215] Fang Y, Fullwood MJ (2016). Roles, Functions, and Mechanisms of Long Non-coding RNAs in Cancer. Genomics Proteomics Bioinformatics.

[216] Cesana M, Cacchiarelli D, Legnini I, Santini T, Sthandier O, Chinappi M (2011). A long noncoding RNA controls muscle differentiation by functioning as a competing endogenous RNA. Cell.

[217] Kung JT, Colognori D, Lee JT (2013). Long noncoding RNAs: past, present, and future. Genetics.

[218] Wilusz JE, Sunwoo H, Spector DL (2009). Long noncoding RNAs: functional surprises from the RNA world. Genes Dev.

[219] Malka Y, Steiman-Shimony A, Rosenthal E, Argaman L, Cohen-Daniel L, Arbib E (2017). Post-transcriptional 3 -UTR cleavage of mRNA transcripts generates thousands of stable uncapped autonomous RNA fragments. Nat Commun.

[220] Mauer J, Luo X, Blanjoie A, Jiao X, Grozhik AV, Patil DP (2017). Reversible methylation of m(6)Am in the 5' cap controls mRNA stability. Nature.

[221] Li JH, Liu S, Zhou H, Qu LH, Yang JH (2014). starBase v2.0: decoding miRNA-ceRNA, miRNA-ncRNA and protein-RNA interaction networks from large-scale CLIP-Seq data. Nucleic Acids Res.

[222] Roundtree IA, Evans ME, Pan T, He C (2017). Dynamic RNA Modifications in Gene Expression Regulation. Cell.

[223] Alarcon CR, Lee H, Goodarzi H, Halberg N, Tavazoie SF (2015). N6-methyladenosine marks primary microRNAs for processing. Nature.

[224] Konno M, Koseki J, Asai A, Yamagata A, Shimamura T, Motooka D (2019). Distinct methylation levels of mature microRNAs in gastrointestinal cancers. Nat Commun.

[225] Wang JX, Gao J, Ding SL, Wang K, Jiao JQ, Wang Y (2015). Oxidative Modification of miR-184 Enables It to Target Bcl-xL and Bcl-w. Mol Cell.

[226] Shin C, Nam JW, Farh KK, Chiang HR, Shkumatava A, Bartel DP (2010). Expanding the microRNA targeting code: functional sites with centered pairing. Mol Cell.

[227] Moretti F, Thermann R, Hentze MW (2010). Mechanism of translational regulation by miR-2 from sites in the 5' untranslated region or the open reading frame. RNA.

[228] Ha I, Wightman B, Ruvkun G (1996). A bulged lin-4/lin-14 RNA duplex is sufficient for Caenorhabditis elegans lin-14 temporal gradient formation. Genes Dev.

[229] Vella MC, Choi EY, Lin SY, Reinert K, Slack FJ (2004). The C. elegans microRNA let-7 binds to imperfect let-7 complementary sites from the lin-41 3'UTR. Genes Dev.

[230] Lal A, Navarro F, Maher CA, Maliszewski LE, Yan N, O'Day E (2009). miR-24 Inhibits cell proliferation by targeting E2F2, MYC, and other cell-cycle genes via binding to "seedless" 3'UTR microRNA recognition elements. Mol Cell.

[231] Zhang K, Zhang X, Cai Z, Zhou J, Cao R, Zhao Y (2018). A novel class of microRNA-recognition elements that function only within open reading frames. Nat Struct Mol Biol.

[232] Tay Y, Zhang J, Thomson AM, Lim B, Rigoutsos I (2008). MicroRNAs to Nanog, Oct4 and Sox2 coding regions modulate embryonic stem cell differentiation. Nature.

[233] Licatalosi DD, Mele A, Fak JJ, Ule J, Kayikci M, Chi SW (2008). HITS-CLIP yields genome-wide insights into brain alternative RNA processing. Nature.

[234] Ule J, Jensen KB, Ruggiu M, Mele A, Ule A, Darnell RB (2003). CLIP identifies Nova-regulated RNA networks in the brain. Science.

[235] Chi SW, Zang JB, Mele A, Darnell RB (2009). Argonaute HITS-CLIP decodes microRNA-mRNA interaction maps. Nature.

[236] Bottini S, Pratella D, Grandjean V, Repetto E, Trabucchi M (2018). Recent computational developments on CLIP-seq data analysis and microRNA targeting implications. Brief Bioinform.

[237] Loeb GB, Khan AA, Canner D, Hiatt JB, Shendure J, Darnell RB (2012). Transcriptome-wide miR-155 binding map reveals widespread noncanonical microRNA targeting. Mol Cell.

[238] Matkovich SJ, Van Booven DJ, Eschenbacher WH, Dorn GW 2nd (2011). RISC RNA sequencing for context-specific identification of *in vivo* microRNA targets. Circ Res.

[239] Seok H, Ham J, Jang ES, Chi SW (2016). MicroRNA Target Recognition: Insights from Transcriptome-Wide Non-Canonical Interactions. Mol Cells.

[240] Travis AJ, Moody J, Helwak A, Tollervey D, Kudla G (2014). Hyb: a bioinformatics pipeline for the analysis of CLASH (crosslinking, ligation and sequencing of hybrids) data. Methods.

[241] Helwak A, Kudla G, Dudnakova T, Tollervey D (2013). Mapping the human miRNA interactome by CLASH reveals frequent noncanonical binding. Cell.

[242] Hafner M, Landthaler M, Burger L, Khorshid M, Hausser J, Berninger P (2010). Transcriptome-wide identification of RNA-binding protein and microRNA target sites by PAR-CLIP. Cell.

[243] Schnall-Levin M, Zhao Y, Perrimon N, Berger B (2010). Conserved microRNA targeting in Drosophila is as widespread in coding regions as in 3'UTRs. Proc Natl Acad Sci U S A.

[244] Liu G, Zhang R, Xu J, Wu CI, Lu X (2015). Functional conservation of both CDS- and 3'-UTR-located microRNA binding sites between species. Mol Biol Evol.

[245] Gu S, Jin L, Zhang F, Sarnow P, Kay MA (2009). Biological basis for restriction of microRNA targets to the 3' untranslated region in mammalian mRNAs. Nat Struct Mol Biol.

[246] Hausser J, Syed AP, Bilen B, Zavolan M (2013). Analysis of CDS-located miRNA target sites suggests that they can effectively inhibit translation. Genome Res.

[247] Grosswendt S, Filipchyk A, Manzano M, Klironomos F, Schilling M, Herzog M (2014). Unambiguous identification of miRNA:target site interactions by different types of ligation reactions. Mol Cell.

[248] Spengler RM, Zhang X, Cheng C, McLendon JM, Skeie JM, Johnson FL (2016). Elucidation of transcriptome-wide microRNA binding sites in human cardiac tissues by Ago2 HITS-CLIP. Nucleic Acids Res.

[249] Broughton JP, Pasquinelli AE (2013). Identifying Argonaute binding sites in Caenorhabditis elegans using iCLIP. Methods.

[250] Mittal N, Zavolan M (2014). Seq and CLIP through the miRNA world. Genome Biol.

[251] Sehgal A, Chen Q, Gibbings D, Sah DW, Bumcrot D (2014). Tissue-specific gene silencing monitored in circulating RNA. RNA.

[252] Sardh E, Harper P, Balwani M, Stein P, Rees D, Bissell DM (2019). Phase 1 Trial of an RNA Interference Therapy for Acute Intermittent Porphyria. N Engl J Med.

[253] Chan A, Liebow A, Yasuda M, Gan L, Racie T, Maier M (2015). Preclinical Development of a Subcutaneous ALAS1 RNAi Therapeutic for Treatment of Hepatic Porphyrias Using Circulating RNA Quantification. Mol Ther Nucleic Acids.

[254] Gallant-Behm CL, Piper J, Lynch JM, Seto AG, Hong SJ, Mustoe TA (2019). A MicroRNA-29 Mimic (Remlarsen) Represses Extracellular Matrix Expression and Fibroplasia in the Skin. J Invest Dermatol.

[255] Wang Y, Liu J, Chen J, Feng T, Guo Q (2015). MiR-29 mediates TGFbeta 1-induced extracellular matrix synthesis through activation of Wnt/beta -catenin pathway in human pulmonary fibroblasts. Technol Health Care.

[256] Daige CL, Wiggins JF, Priddy L, Nelligan-Davis T, Zhao J, Brown D (2014). Systemic delivery of a miR34a mimic as a potential therapeutic for liver cancer. Mol Cancer Ther.

[257] Hong DS, Kang YK, Borad M, Sachdev J, Ejadi S, Lim HY (2020). Phase 1 study of MRX34, a liposomal miR-34a mimic, in patients with advanced solid tumours. Br J Cancer.

[258] Vafaee F, Diakos C, Kirschner MB, Reid G, Michael MZ, Horvath LG (2018). A data-driven, knowledge-based approach to biomarker discovery: application to circulating microRNA markers of colorectal cancer prognosis. NPJ Syst Biol Appl.

[259] Hatz S, Spangler S, Bender A, Studham M, Haselmayer P, Lacoste AMB (2019). Identification of pharmacodynamic biomarker hypotheses through literature analysis with IBM Watson. PLoS One.

[260] Zuckerman JE, Davis ME (2015). Clinical experiences with systemically administered siRNA-based therapeutics in cancer. Nat Rev Drug Discov.

[261] Titze-de-Almeida R, David C, Titze-de-Almeida SS (2017). The Race of 10 Synthetic RNAi-Based Drugs to the Pharmaceutical Market. Pharm Res.

[262] Szallasi A, Cortright DN, Blum CA, Eid SR (2007). The vanilloid receptor TRPV1: 10 years from channel cloning to antagonist proof-of-concept. Nat Rev Drug Discov.

[263] Benitez-Del-Castillo JM, Moreno-Montanes J, Jimenez-Alfaro I, Munoz-Negrete FJ, Turman K, Palumaa K, Sadaba B (2016). Safety and Efficacy Clinical Trials for SYL1001, a Novel Short Interfering RNA for the Treatment of Dry Eye Disease. Invest Ophthalmol Vis Sci.

[264] Weinreb RN, Aung T, Medeiros FA (2014). The pathophysiology and treatment of glaucoma: a review. JAMA.

[265] Moreno-Montanes J, Sadaba B, Ruz V, Gomez-Guiu A, Zarranz J, Gonzalez MV (2014). Phase I clinical trial of SYL040012, a small interfering RNA targeting beta-adrenergic receptor 2, for lowering intraocular pressure. Mol Ther.

[266] Vigneswara V, Ahmed Z (2016). Long-term neuroprotection of retinal ganglion cells by inhibiting caspase-2. Cell Death Discov.

[267] Nguyen QD, Schachar RA, Nduaka CI, Sperling M, Basile AS, Klamerus KJ (2012). Phase 1 dose-escalation study of a siRNA targeting the RTP801 gene in age-related macular degeneration patients. Eye.

[268] Molitoris BA, Dagher PC, Sandoval RM, Campos SB, Ashush H, Fridman E (2009). siRNA targeted to p53 attenuates ischemic and cisplatin-induced acute kidney injury. J Am Soc Nephrol.

[269] Tabernero J, Shapiro GI, LoRusso PM, Cervantes A, Schwartz GK, Weiss GJ (2013). First-in-humans trial of an RNA interference therapeutic targeting VEGF and KSP in cancer patients with liver involvement. Cancer Discov.

[270] Carmeliet P (2005). VEGF as a key mediator of angiogenesis in cancer. Oncology.

[271] Tang PA, Siu LL, Chen EX, Hotte SJ, Chia S, Schwarz JK (2008). Phase II study of ispinesib in recurrent or metastatic squamous cell carcinoma of the head and neck. Invest New Drugs.

[272] Gutteridge RE, Ndiaye MA, Liu X, Ahmad N (2016). Plk1 Inhibitors in Cancer Therapy: From Laboratory to Clinics. Mol Cancer Ther.

[273] El Dika I, Lim HY, Yong WP, Lin CC, Yoon JH, Modiano M (2019). An Open-Label, Multicenter, Phase I, Dose Escalation Study with Phase II Expansion Cohort to Determine the Safety, Pharmacokinetics, and Preliminary Antitumor Activity of Intravenous TKM-080301 in Subjects with Advanced Hepatocellular Carcinoma. Oncologist.

[274] Schultheis B, Strumberg D, Santel A, Vank C, Gebhardt F, Keil O (2014). First-in-human phase I study of the liposomal RNA interference therapeutic Atu027 in patients with advanced solid tumors. J Clin Oncol.

[275] Zorde Khvalevsky E, Gabai R, Rachmut IH, Horwitz E, Brunschwig Z, Orbach A (2013). Mutant KRAS is a druggable target for pancreatic cancer. Proc Natl Acad Sci U S A.

[276] Golan T, Khvalevsky EZ, Hubert A, Gabai RM, Hen N, Segal A (2015). RNAi therapy targeting KRAS in combination with chemotherapy for locally advanced pancreatic cancer patients. Oncotarget.

[277] Grimm D, Kay MA (2007). Combinatorial RNAi: a winning strategy for the race against evolving targets?. Mol Ther.

[278] Hudgin RL, Pricer WE Jr, Ashwell G, Stockert RJ, Morell AG (1974). The isolation and properties of a rabbit liver binding protein specific for asialoglycoproteins. J Biol Chem.

[279] Steer CJ, Ashwell G (1980). Studies on a mammalian hepatic binding protein specific for asialoglycoproteins. Evidence for receptor recycling in isolated rat hepatocytes. J Biol Chem.

[280] Ines M, Coelho T, Conceicao I, Duarte-Ramos F, de Carvalho M, Costa J (2018). Epidemiology of Transthyretin Familial Amyloid Polyneuropathy in Portugal: A Nationwide Study. Neuroepidemiology.

[281] Saraiva MJ (2002). Hereditary transthyretin amyloidosis: molecular basis and therapeutical strategies. Expert Rev Mol Med.

[282] Coelho T, Adams D, Silva A, Lozeron P, Hawkins PN, Mant T (2013). Safety and efficacy of RNAi therapy for transthyretin amyloidosis. N Engl J Med.

[283] Nordmann Y, Puy H, Da Silva V, Simonin S, Robreau AM, Bonaiti C (1997). Acute intermittent porphyria: prevalence of mutations in the porphobilinogen deaminase gene in blood donors in France. J Intern Med.

[284] Kauppinen R (2005). Porphyrias. Lancet.

[285] Peterson AS, Fong LG, Young SG (2008). PCSK9 function and physiology. J Lipid Res.

[286] Fitzgerald K, White S, Borodovsky A, Bettencourt BR, Strahs A, Clausen V (2017). A Highly Durable RNAi Therapeutic Inhibitor of PCSK9. N Engl J Med.

[287] Janssen HL, Reesink HW, Lawitz EJ, Zeuzem S, Rodriguez-Torres M, Patel K (2013). Treatment of HCV infection by targeting microRNA. N Engl J Med.

[288] van Rooij E, Sutherland LB, Thatcher JE, DiMaio JM, Naseem RH, Marshall WS (2008). Dysregulation of microRNAs after myocardial infarction reveals a role of miR-29 in cardiac fibrosis. Proc Natl Acad Sci U S A.

[289] Liu Y, Taylor NE, Lu L, Usa K, Cowley AW Jr, Ferreri NR (2010). Renal medullary microRNAs in Dahl salt-sensitive rats: miR-29b regulates several collagens and related genes. Hypertension.

[290] Calin GA, Dumitru CD, Shimizu M, Bichi R, Zupo S, Noch E (2002). Frequent deletions and down-regulation of micro- RNA genes miR15 and miR16 at 13q14 in chronic lymphocytic leukemia. Proc Natl Acad Sci U S A.

[291] Cimmino A, Calin GA, Fabbri M, Iorio MV, Ferracin M, Shimizu M (2005). miR-15 and miR-16 induce apoptosis by targeting BCL2. Proc Natl Acad Sci U S A.

[292] Liu Q, Fu H, Sun F, Zhang H, Tie Y, Zhu J (2008). miR-16 family induces cell cycle arrest by regulating multiple cell cycle genes. Nucleic Acids Res.

[293] Calin GA, Cimmino A, Fabbri M, Ferracin M, Wojcik SE, Shimizu M (2008). MiR-15a and miR-16-1 cluster functions in human leukemia. Proc Natl Acad Sci U S A.

[294] MacDiarmid JA, Mugridge NB, Weiss JC, Phillips L, Burn AL, Paulin RP (2007). Bacterially derived 400 nm particles for encapsulation and cancer cell targeting of chemotherapeutics. Cancer Cell.

[295] van Zandwijk N, Pavlakis N, Kao SC, Linton A, Boyer MJ, Clarke S (2017). Safety and activity of microRNA-loaded minicells in patients with recurrent malignant pleural mesothelioma: a first-in-man, phase 1, open-label, dose-escalation study. Lancet Oncol.

[296] Slabakova E, Culig Z, Remsik J, Soucek K (2017). Alternative mechanisms of miR-34a regulation in cancer. Cell Death Dis.

[297] Misso G, Di Martino MT, De Rosa G, Farooqi AA, Lombardi A, Campani V (2014). Mir-34: a new weapon against cancer?. Mol Ther Nucleic Acids.

[298] Wang X, Li J, Dong K, Lin F, Long M, Ouyang Y (2015). Tumor suppressor miR-34a targets PD-L1 and functions as a potential immunotherapeutic target in acute myeloid leukemia. Cell Signal.

[299] Wiggins JF, Ruffino L, Kelnar K, Omotola M, Patrawala L, Brown D (2010). Development of a lung cancer therapeutic based on the tumor suppressor microRNA-34. Cancer Res.

[300] Cortez MA, Ivan C, Valdecanas D, Wang X, Peltier HJ, Ye Y (2016). PDL1 Regulation by p53 via miR-34. J Natl Cancer Inst.

[301] Wu J, Li X, Li D, Ren X, Li Y, Herter EK (2020). MicroRNA-34 Family Enhances Wound Inflammation by Targeting LGR4. J Invest Dermatol.

[302] Postow MA, Callahan MK, Wolchok JD (2015). Immune Checkpoint Blockade in Cancer Therapy. J Clin Oncol.

[303] Shi XB, Xue L, Yang J, Ma AH, Zhao J, Xu M (2007). An androgen-regulated miRNA suppresses Bak1 expression and induces androgen-independent growth of prostate cancer cells. Proc Natl Acad Sci U S A.

[304] Cevec M, Thibaudeau C, Plavec J (2008). Solution structure of a let-7 miRNA:lin-41 mRNA complex from C. elegans. Nucleic Acids Res.

[305] Lee I, Ajay SS, Yook JI, Kim HS, Hong SH, Kim NH (2009). New class of microRNA targets containing simultaneous 5'-UTR and 3'-UTR interaction sites. Genome Res.

